# Bridging behavioral theory and household energy decisions: enhancing agent-based models with behavioral analysis

**DOI:** 10.3389/fpsyg.2025.1568730

**Published:** 2025-07-09

**Authors:** Mariëlle Rietkerk-van der Wijngaart, Lynn de Jager, Geeske Scholz, Emile Chappin, Gerdien de Vries

**Affiliations:** ^1^Faculty of Technology, Policy and Management, Delft University of Technology (TU Delft), Delft, Netherlands; ^2^Centre for Sustainability, Environment and Health, National Institute for Public Health and the Environment (RIVM), Bilthoven, Netherlands

**Keywords:** agent-based models, household decision, energy transition, behavioral insights, policy design

## Abstract

Households are crucial in the energy transition, accounting for over 25% of the European Union's energy consumption. To design effective policy measures that motivate households to change their behavior in favor of the energy transition, agent-based models (ABMs) are vital. For ABMs to reach their full potential in policy design, they must appropriately represent behavioral dynamics. One way to accomplish this is by strengthening the fit in ABMs between behavioral determinants (e.g., trust in energy companies) and the behavior of interest (e.g., adopting tariff structures). This study investigates whether a structured behavioral analysis improves this “determinants-behavior-fit.” A systematic review of 71 ABMs addressing household energy decisions reveals that models incorporating a behavioral analysis formalize nearly twice as many behavioral determinants, indicating a more systematic uptake. Subsequently, we find a difference between models focusing on investment-related behaviors (e.g., households buying solar panels) and those examining daily energy practices (e.g., households adjusting charging habits). Models in the first category integrate more social factors when incorporating behavioral analyses, corresponding with the influence of networks and peer effects on investment behaviors. Models in the second category emphasize individual and external factors in response to behavioral analyses, corresponding with the energy practices' habitual and contextual nature. Despite the benefits of a behavioral analysis for improving the determinants-behavior fit in ABMs, only one-third of the studies apply it partially. On top of that, almost half of the studies do not report a rationale for their choice of behavioral determinants. This suggests that many models may not fully capture the behavioral mechanisms underlying household energy decisions, limiting ABMs' potential to inform policymakers. Our findings highlight the need for systematic behavioral assessments in model development. We conclude that collaboration between behavioral scientists and modelers is crucial to accomplish such integration, and we emphasize the importance of allowing sufficient time and resources for meaningful exchange. Future research could further investigate empirical validation of behavioral insights in ABMs and explore how ABM results improve with a better determinants-behavior fit. By bridging behavioral science with computational modeling, ABMs' decision-support power to policymakers can be improved, ultimately accelerating the energy transition.

## 1 Introduction

In 2023, global average temperatures have reached nearly 1.5°C above pre-industrial levels (World Meteorological Organisation, 2024), threatening the Paris Agreement's goal to limit this century's global warming to well below 2°C (United Nations, 2015). The energy transition aims to reduce global warming by replacing fossil fuels with renewable energy sources (e.g., Smil, 2010). With households accounting for more than 25% of the final energy consumption in the European Union alone (Eurostat, 2022), governments seek to stimulate households toward more efficient and sustainable energy use.

To stimulate climate-positive actions, behavioral insights have increasingly informed energy policy design in the past decade. It is recognized more and more that behavior is not solely rational or economical but is also driven by cognitive biases, social norms, emotions, and other psychological and contextual factors (Cabinet Office, 2013; Freschi et al., 2023; OECD Organisation for Economic Co-operation Development, 2017). Typically, behavioral science-based policies are more effective because they (1) align better with real-world decision-making, (2) are usually less restricting or coercive and therefore more socially acceptable, (3) are less complex to understand, and (4) allow for a more personalized approach (e.g., Benartzi et al., 2017; Gopalan and Pirog, 2017; Hummel and Maedche, 2019; Troussard and van Bavel, 2018; Thaler and Sunstein, 2009).

However, if policies are solely informed by behavioral insights, the system-level context can be overlooked (Chater and Loewestein, 2022; Steg, 2023), and perspectives on the relationship between psychology and the broader system can remain underdeveloped (De Vries et al., 2021; Freschi et al., 2023). Considering system-level insights for policy design is crucial for two reasons: (1) system processes influence individual decision-making, and (2) unpredictable system-level effects may emerge from individual decisions. First, neglecting the influence of system processes on human behavior can significantly reduce the effectiveness of policies that target individual decision-making. For example, uncertainties related to subsidies and reliance on municipal heat planning can lead homeowners to hesitate in investing in sustainable heating technologies, illustrating how complex market and policy dynamics may unintentionally hinder individual behavior. Second, individual decision-making may lead to the emergence of unpredictable effects at a macro level. The case of CO_2_ storage in the Dutch village of Barendrecht illustrates emergence through the interaction of multiple stakeholders, leading to an unforeseen outcome (Brunsting et al., 2011; Chappin and Blomme, 2022). While research initially found no objections to the project, opposition from residents emerged as the project progressed. This resistance was further amplified by municipal authorities and the local province, ultimately leading to the project's cancellation. Simulating how concerns spread in a social network and identifying feedback loops that amplified the opposition could have helped to anticipate resistance and enhance the effectiveness of policies.

A technique well suited to exploring the interaction of system-level effects with individual decision-making is agent-based modeling (ABM). ABM excels in capturing macro-level system phenomena emerging from micro-level decisions of individual agents like households. It does so by being capable of modeling for interactions of processes on both the individual and the system level. Unlike other modeling techniques, ABM allows for the modeling of heterogeneous agents: different individuals or components in the model can each have unique decision styles representing the diversity of real-world actors. ABM has increasingly been applied in energy transition studies thanks to the method's ability to represent complexity (see, for instance, Moglia et al., 2017, who compare different modeling techniques for studying household energy technology).

However, the utilization of insights from behavioral science in ABMs can be improved. While formalizations of behavioral determinants play a crucial role in shaping model behaviors and the complexity of human behavior is widely acknowledged, agents in ABMs are currently still often driven by simple and rational decision rules (e.g., Schwarz et al., 2020; Senkpiel et al., 2020; Wijermans et al., 2023). Put differently, the fit between formalized behavioral theories and simulated behavior can be strengthened in ABMs.

One way of strengthening this fit is by performing a behavioral analysis (BA). A behavioral analysis is a structured field scan of the behavior of interest. It focuses on understanding why the behavior occurs, how it can be changed, and which influencing behavioral factors are important. A BA is important because behavioral theories are not simply a “one size fits all” solution: different behaviors are influenced by different behavioral factors and theories. We elaborate on the BA-approach in the next chapter.

For ABMs to grow as a decision-support tool for policymakers, a deeper understanding of how behavioral insights have been applied in ABMs is needed. Several reviews have researched the uptake of behavioral insights in energy system modeling. They focus on adopting one certain energy technology, applying one specific behavioral theory in ABMs, and the uptake of behavioral insights in energy policies. They have assessed (a) the range, (b) the degree of simplicity, and (c) the degree of recoverability (if others could access the exact formalizations) of formalized behavioral theories. They concluded that (a) merely a narrow set of possible and suitable psychological constructs is formalized into an agent's decision style (e.g., Castro et al., 2020; Hansen et al., 2019; Hesselink and Chappin, 2019; Huckebrink and Bertsch, 2021; Nurwidiana et al., 2021), (b) that ABMs typically make use of *ad-hoc*, simple and domain-unspecific decision rules (e.g., Akhatova et al., 2022; Du et al., 2022), and (c) that these decisions rules are often not reported in detail (Akhatova et al., 2022) or are difficult to identify (Ribeiro-Rodrigues and Bortoleto, 2024).

Current reviews do not assess the uptake of behavioral insights in ABMs covering the whole range of household energy decisions, including household energy technology use. They also do not review if these behavioral insights are required by utilizing a behavioral analysis (BA). To address this knowledge gap, we focus on the whole domain of household energy decisions, including daily energy practices, and investigate whether the BA method is applied to ABM design. Additionally, we assess the reasoning behind choosing a behavioral theory and whether the BA method improves the fit of behavioral theory with simulated energy behavior.

The following three research questions are formulated. As mentioned above, we assess all ABMs that simulate household energy decisions.

- RQ1: Which behaviors, behavioral factors, and theories (BFTs) are studied?
- RQ2: Is the reasoning for choosing BFTs stated clearly, and does a behavioral analysis back up the reasoning?
- RQ3: Does the fit of BFTs with the modeled behavior improve when a behavioral analysis informs the choice of these BFTs?

The remainder of the paper is organized as follows: In Chapter 2, we position this work concerning current literature and introduce the BA method tailored to model design. The search method and labeling strategy are described in Chapter 3, followed by the results of our review in Chapter 4. A discussion and outlook for further research are presented in Chapter 5. Chapter 6 concludes our findings and recommends how the practice of behavioral analysis can best be integrated into agent-based energy system modeling.

## 2 Behavioral analysis

To understand the potential of behavioral analysis for agent-based modeling, we outline below how an optimized fit of behavioral determinants with simulated behavior in ABMs contributes to more effective policymaking (Sections 2.1 and 2.2). We then introduce how the method of informing policy with behavioral analysis can be translated to the agent-based modeling domain in Section 2.3.

### 2.1 Bridging behavioral theory and simulated energy behavior

Implementing behavioral factors and theories in ABM is critical for designing effective energy policies because it leads to more realistic model outcomes. As Jager (2021) concludes, implementing behavioral insights in ABM comes with the challenge of “*capturing relevant behavioral processes in a valid manner*.” The modelers' choice of a behavioral theory is crucial for this validity (see also Wijermans et al., 2023). Without a good fit of behavioral theory with the behavior addressed in the ABM, the models' validity decreases, and the decision-support power of ABMs to inform energy policy design is reduced significantly.

The “determinant-behavior-fit” is defined as how behavioral factors and theories align with the simulated household energy behavior. We draw heavily on Wijermans et al. (2023), who first put forward the notion of fit as aligning agents' decision context with an agent's behavior. This principle of fit is important because not every behavioral factor or theory can describe each behavior equally well. Take, for example, two very different behaviors: laundry load shifting (where a laundry practice is shifted to another part of the day to avoid overload of the electricity grid) and the adoption of solar panels (where households invest in solar panels on their roof). Doing the laundry is habitual, while buying solar panels is more reflective. Behavioral factors and theories that describe habitual and reflective behaviors differ in how they account for cognitive effort, the processing of new information, and reacting to cues from the direct environment. Therefore, we expect that ABMs that simulate habitual behaviors implement different behavioral factors and theories than ABMs that simulate reflective behaviors.

### 2.2 Behavioral analysis in policy design

For policy design, the notion that the integration of relevant behavioral factors benefits policy outcomes is not new. Already in 2012, the European Commission launched a “Summer School” in behavioral economics for EU policymakers driven by the belief that policymakers can more actively apply behavioral insights to improve policy development (Van Bavel et al., 2013). In 2015, Barack Obama directed all governmental agencies to incorporate behavioral insights into policy design (White House Press Secretary Office, 2015). Shortly after, a centralized team (the “social and behavioral sciences team”) was established, which began to identify behavioral precursors and to conduct empirical policy testing on a large scale.

Scientific literature also shows that implementing psychological factors that originate from careful consideration leads to policy tools that better motivate behavior change (e.g., in the context of market failure, Madrian, 2014). Gopalan and Pirog (2017) proposed a decision framework for policymakers to integrate behavioral insights, including exploring psychological impediments that preclude behavior. Similarly, Kuehnhanss (2019) emphasizes the need for careful consideration of behavioral factors in policy design. He argues that integrating behavioral insights into policymaking requires not only translating academic findings into the political and administrative process, but also ensuring that policymakers and institutions have the necessary skills and capacity to apply them effectively. The textbook “Behavioral Insights for Policy Design” (Lichand et al., 2023) aims to educate policymakers in this field. It seeks to achieve the “*inclusion of broader and deeper insights from the behavioral sciences […] by introducing new methodologies for diagnosing the root causes behind public problems and for designing effective policies to address them.”*

When implementing behavioral insights in policy design, three types of behavioral policy initiatives can be classified, defined by the degree to which behavioral considerations have helped shape them: aligned, informed, and tested (Sousa Lourenço et al., 2016). First, behavioral-aligned initiatives are those that, in hindsight, align with behavioral science principles, but they are not explicitly based on findings from research or trials. They incorporate behavioral insights such as using framing effects or leveraging loss aversion (e.g., presenting 20% fat cheese as 80% fat-free or implementing decremental penalty points for driving licenses). Second, behavioral-informed initiatives are deliberately designed using established behavioral evidence, though they have not been tested in specific contexts (e.g., banning pre-checked boxes or introducing standardized tobacco packaging). Third, the highest degree of behavioral consideration can be found in the behavioral-tested initiatives, which are grounded in behavioral science and refined through experiments before being widely implemented. An overview of energy policy initiatives in the third (behaviorally-tested) category can be found in the “environment scan” that was produced in commission of the International Energy Agency (UsersTCP IEA., 2020). Based on the scan, the authors designed a tool (see https://bitoolkit.userstcp.org/index.html) to help policymakers apply behavioral insights. The tool leads the user through a set of choices (e.g., if they are developing a new program, if there is a desired policy instrument, and if the policy regards the uptake of new technology or a change in consumption pattern). It then returns a list of behavioral-tested factors that affect the success of an energy policy.

To arrive at behavioral-informed or -tested policies, conducting behavioral analyses prior to policy design is becoming increasingly common to improve policy interventions aimed at influencing energy behaviors. The debate has shifted from whether policymakers should apply a behavioral analysis to the more practical questions of where, when, and how they should be integrated into the policy process (Baggio et al., 2021). A behavioral analysis typically involves two stages: identifying the behavioral challenge and analyzing its root causes and influences (Baggio et al., 2021; Troussard and van Bavel, 2018). According to the European Commission's Better Regulation framework (a set of principles guiding the development, implementation, and evaluation of policies; European Commission, 2021), a behavioral analysis is best conducted in the early stages of policy design.

### 2.3 The behavioral analysis approach for ABM design

**Figure 1:**
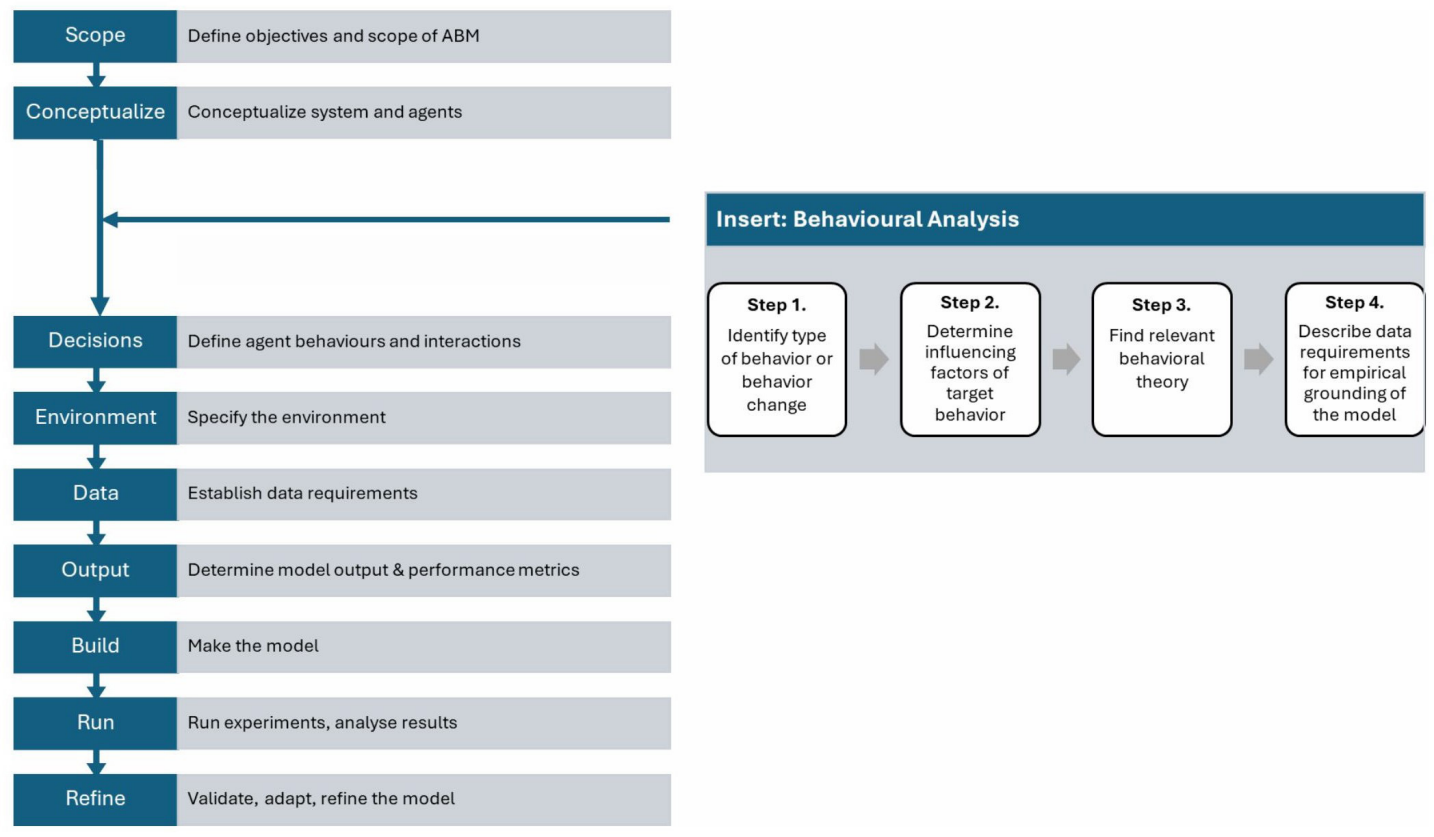
Flowchart depicting a process for defining an agent-based model (ABM) and behavioral analysis. The left column lists the steps: Scope, Conceptualize, Decisions, Environment, Data, Output, Build, Run, and Refine. The right section titled “Insert: Behavioural Analysis” includes four steps: identifying behavior types, determining influencing factors, finding theories, and describing data requirements. Arrows indicate the progression and connections between steps. Incorporating the four-step behavioral analysis approach in the workflow of ABM design.

Based on the literature on how behavioral insights are integrated into policy design, we introduce a behavioral analysis (BA) approach tailored to agent-based modeling. We shortly outline four steps in conducting a behavioral analysis below: (1) identifying the type of behavior and/or behavior change, (2) examining the influencing factors of behavior, (3) finding relevant theories that explain behavior, and (4) describing data collection requirements for empirically grounding the model. We have depicted the four steps of the behavioral analysis in Figure 1, suggesting its place in the workflow of designing an ABM [we adapted the ABM workflow from Macal and North (2010)]. Because ABMs draw heavily on behavioral rules, we recommend that a behavioral analysis is performed at the early stages of designing a model (Figure 1).

The first step of a BA entails identifying the relevant behavior change with precision. What is the current behavior, and what is the target behavior? Does the behavior need to start, stop, or only be adjusted? Is the focus on reinforcing the target behavior or on reducing the current behavior? What is the current status quo of these behaviors in the population that you want to investigate? These questions typically lead to articulating intervention goals tailored for different target groups.

For step one, it is important to note that behavior includes everything that one or more actors do that is observable and measurable (e.g., Uher, 2016). This is in contrast with definitions of behavior that include “introspectively observable activities and non-conscious processes” (American Psychological Association, 2018).

Observable means that behavior can be seen by the naked eye. For example, a smile can be observed, but emotional states like joy or happiness cannot be directly seen. Measurable means that behavior can be quantified directly: how many times, how often, and how long a behavior takes place can be measured. For example, getting mad cannot be quantified directly, but slamming doors can. Defining behavior like this is important for empirical validation, because it provides a boundary between what can be objectively studied and what remains interpretative between researchers. In ABMs, an example that typically occurs is the concept of learning. According to the above definition of behavior, learning itself is not marked as behavior but as a cognitive process that leads to learned or unlearned behavior.

*Illustration of step 1*.

*Household load shifting can involve various target behaviors. For example, households may be encouraged to delay charging their electric vehicle after returning home from work, to prevent grid congestion. This target behavior (charging after 9:00 PM) is an adjustment of the current behavior (charging around 6:00 PM). In analyzing the status quo, we could, for instance, find that more than half of Dutch households with electric vehicles charge their cars upon arrival at home in the evening. Based on this, we could focus our behavioral analysis on households with day jobs in another city and who commute by car*.

The second step of a BA is to identify the factors that influence the behavior in question. These factors are individual or external by nature: they happen within or outside a person. As individual factors are formulated in the mind, they include processes that influence mental states and how someone perceives and processes information (Reynolds and Miller, 2015). Examples of individual factors are emotions, attitudes, lifestyle, stress, demographics, and personality. External factors to behavior, on the other hand, relate to the situation or circumstances in which behavior is formed; they refer to the environment in which information is processed. External factors are typically divided into technological (e.g., noise or location of technology), economic (e.g., price of products), institutional (e.g., fiscal policies), environmental (e.g., weather conditions), and social circumstances (e.g., cultural norms). See, for example, Perlaviciute and Steg (2014), who give an overview of how individual and external factors influence public attitudes about large-scale energy technology.

For step two, the influencing factors must be specifically aligned with the behavior being studied in the model. The fit between behavioral determinants and behavior must be strong, as individual and external drivers shaping one behavior may not meaningfully translate to another. For example, when modeling household heat pump adoption, evaluating the behavioral factors that influence the acceptance of wind farms would not be very informative: if a population's distribution of “attitudes to wind farms” is implemented in a model of heat pump adoption, this would create little insight. Models could misrepresent key influences without ensuring that behavioral factors are specific to the behavior in question, resulting in ineffective policy recommendations.

*Illustration of step 2*.

*In this step, we review the literature on both individual and external factors influencing the target behavior. For example, we identify grey literature describing pilot studies that investigate shifting electric vehicle charging away from peak hours. In these studies, environmental concern and range anxiety were explored as behavioral determinants (internal factors). Additionally, we highlight peer-reviewed literature showing that individuals tend to favor certain tariff structures (external factor) over others in relation to load-shifting practices (Hayn et al., 2018)*.

The third step of a BA is identifying relevant behavioral theories or conceptual frameworks. These describe and predict how specific behaviors are acquired, strengthened, or weakened and how external and individual factors shape them. Theories and frameworks are well-established and validated by experiments and evidence. An example of a behavioral theory is the theory of diffusion of innovation (Rogers, 1962), where several characteristics of innovation (e.g., how complex it is to use the innovation or how compatible the innovation is to the user) result in five types of people: innovators, early adopters, early majority, late majority, and laggards. An example of a conceptual behavioral framework is the Consumat Framework (Jager et al., 2000). Consumat is based on concepts and theories from different scientific domains, and it proposes that behavior is eventually formed through four different cognitive processes: repetition, deliberation, imitation, and social comparison.

In step three, the behavioral analysis explores whether a behavioral theory describes behavior at the macro, micro, or meso level and weighs the pros and cons of formalizing one theory over another. It is crucial to acknowledge that relevant behavioral theories and frameworks are hard to pinpoint because they are abundant and of various sorts. They can, for instance, attempt to factor in all, or at least most, elements leading to behavior, or they can focus on explaining one particular factor (like the concept of biased human decision-making or habit strength). Several attempts have been made to collect social-psychological theories (e.g., Biely, 2022) or to come to a meta-theory of human behavior (e.g., Vallacher and Nowak, 1994). However, these efforts can even make it harder to identify a relevant behavioral theory.

Finding a gap in the literature between empirically researched behavioral factors (step 2 of the behavioral analysis) and suitable theories that explain the behavior of interest could help define the goal of the model. For example, if the Social Identity Theory (Tajfel and Turner, 2001) is found to be relevant for explaining the behavior of interest and literature mainly deals with social factors but not with self-image (two main parts of the theory), the goal of the model could be to explore the impact of self-image on behavior.

*Illustration of step 3*.

*In this step, we review several social science theories, identifying how they can explain our target behavior. Specifically, we highlight the theory of habit formation (Wood and Neal, 2007), which involves cues, routines, and rewards, and social practice theory (Shove, 2003), which emphasizes the evolution of practices through factors such as materials, competence, and meaning. We find that both theories are well-suited to explain household load shifting (Hess et al., 2018; Hubert et al., 2024; Webb et al., 2021) and Furthermore, we describe the goal of our ABM, which is to investigate the extent to which peak demand is reduced in specific districts when households are provided with an app that cues the timing of charging. With this goal in mind, we formalize a framework that integrates both theories, including the element of tariff structures identified in step 2*.

The fourth and last step of a BA is a discussion of the most suitable way of data collection to provide for the empirical grounding of the model. The data collection method largely depends on the current state of knowledge on behavioral factors and theories. Typically, if little is known about a behavioral factor, a qualitative research method is better suited to gain valuable data and insights than a quantitative research method (Bourne et al., 2021).

*Illustration of step 4*.

*In this step, we outline the data gaps identified from our analysis in steps 2 and 3. As a result, we emphasize the need to collect data on current charging routines through a series of interviews. Additionally, we note that while tariff structures have been empirically tested in relation to shifting laundry practices (Hayn et al., 2018), similar data for electric vehicle charging is lacking. To address this, we propose conducting a discrete choice experiment to uncover preferences in tariff structures more relevant to our target behavior*.

In summary, the behavioral analysis (BA) approach provides a structured way to integrate behavioral realism into ABMs by systematically identifying relevant behaviors (step 1), understanding their drivers (step 2), selecting appropriate theoretical foundations (step 3), and outlining data needs for empirical grounding (step 4). This four-step process encourages a deeper reflection on how and why agents behave the way they do, ultimately supporting the development of more robust and policy-relevant ABMs.

## 3 Method

### 3.1 PRISMA-grounded search for studies under review

To obtain a data set of eligible studies for quantitative synthesis, our search methodology was grounded in the framework and guidelines provided by the Preferred Reporting Items for Systematic Reviews and Meta-Analyses (PRISMA) methodology (Moher et al., 2009; Rethlefsen et al., 2021). Studies were identified by explicitly applying a broad search string in the Scopus database to retrieve as many relevant studies as possible. Three main concepts (ABM, household, and energy technology) were entered, and Boolean operators were used to refine the search. The search string is depicted below and was applied on March 1, 2024.

TITLE-ABS (ABM OR “agent-based modeling” OR “agent-based modelling”) AND (“energy efficiency” OR “energy technology”) AND (“household” OR “residential” OR “consumer”)

The search string resulted in a total of 298 empirical papers, including 23 review papers. We screened the abstracts of the 23 review papers, and if ABMs regarding household energy decisions were under investigation, the full text of the review was analyzed, and studies relevant to the analysis were singled out. After including these studies as records in our dataset, the network of experts in our research team was queried for relevant research that matched our criteria.

This resulted in a total of 301 records. After duplicates were removed, all abstracts were screened, and studies were included when (a) an ABM was developed and described, (b) a household decision was likely to be studied, (c) the energy transition is the main focus of the household decision, and (d) the abstract was written in English.

Next, the remaining 117 records were screened for eligibility based on the full text of the article. Only open-access, English-written papers in which household energy decisions were studied using ABM were considered. Papers that covered the modeling of electricity markets, businesses, and offices or papers that modeled household decision-making regarding the circular economy (e.g., the reuse or repair of products) were excluded.

**Figure 2:**
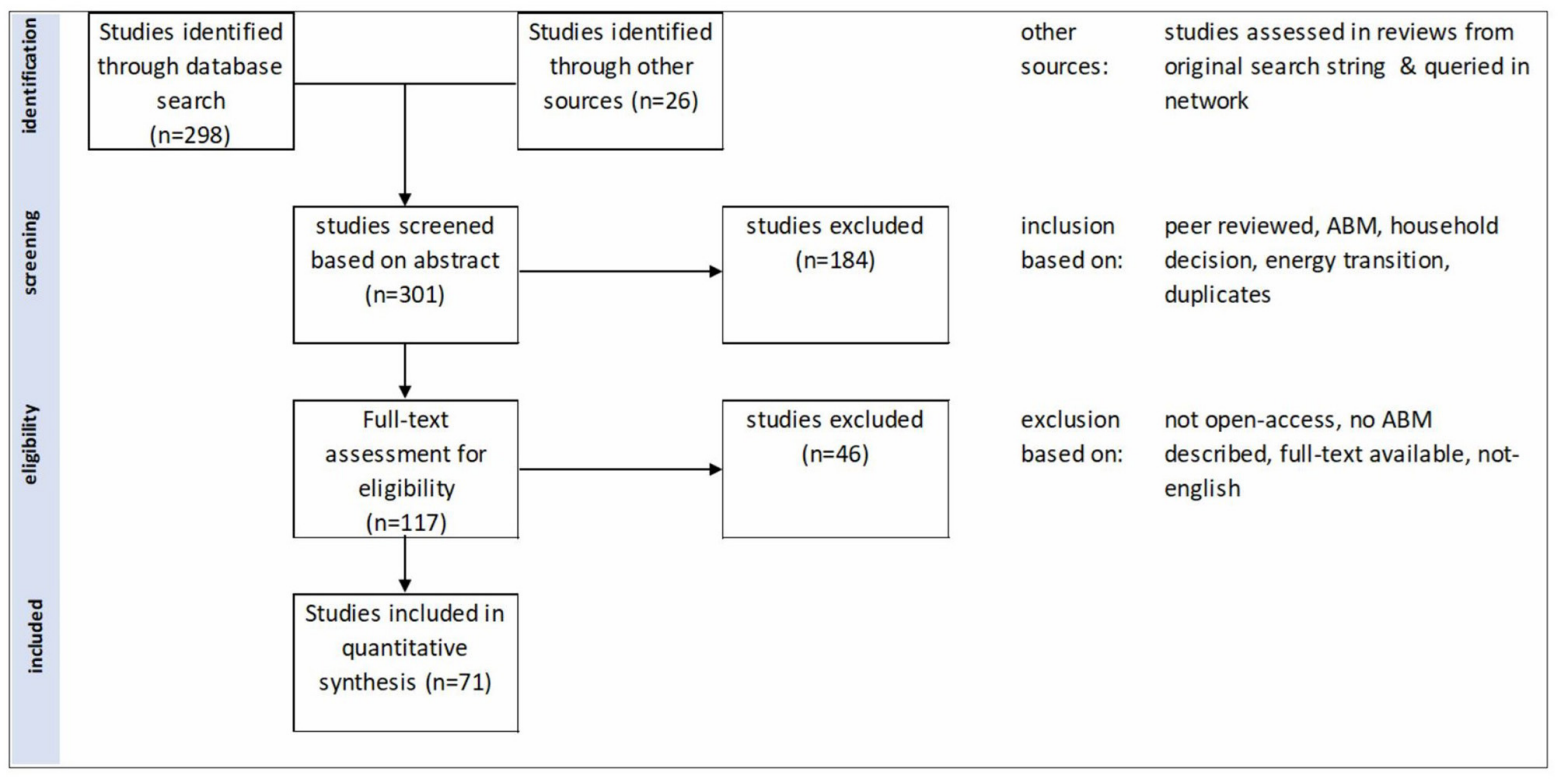
PRISMA flowchart toward the sample of studies used for our review.

After the full-text assessment, a total of 71 records were included for data extraction and analysis. The adapted PRISMA flowchart in Figure 2 displays the protocol followed. Appendix A contains a list of the studies under review. In the data availability statement, we reference a DOI link to the 4TU data repository, in which we provide an Excel file including publications as rows and BFTs, type of behavior, levels of behavior (Adoption or Use), levels of behavioral analysis, and levels of arguments in columns.

### 3.2 Extracting data and structural coding

This review reports findings at the aggregate level of household adoption and use of energy technologies. We do not assess the fit of behavioral factors or theories at the level of individual behaviors simulated within ABMs. In other words, we did not make a priori assumptions about which behavioral factors or theories should be applied to specific behaviors. As such, we do not evaluate whether particular models demonstrate a good or poor fit. In our view, each specific behavior needs a thorough behavioral analysis to reach such a conclusion.

#### 3.2.1 Distinction between Adoption- and Use-ABMs

In this review, we analyze the behavior of households, specifically the interactions that one or more households have with energy technology. These behaviors range from adopting an energy technology (which we label “Adoption-ABMs,” e.g., buying solar panels) to using an energy technology (which we label “Use-ABMs,” e.g., using appliances more efficiently). Acceptance of energy technologies or attitudes and opinions toward an energy technology were not marked as behavior but as factors leading to behavior.

ABMs were labeled as “Adoption-ABMs” when households were expected to buy energy technology (e.g., energy feedback devices like in-home displays or solar panels), when they were to engage in extensive retrofits (e.g., improving the isolation of the house), or when they had to accept a significant change (e.g., agreeing with a connection to district heating). ABMs were labeled as “Use-ABMs” when the operation of an energy technology was the primary focus (e.g., reducing energy demand by lowering thermostat settings), when habits were expected to change (e.g., reacting to feed-in tariffs to shift energy use to another part of the day), or when households were sharing their energy use (e.g., sharing energy from a commonly owned energy source within a neighborhood). Two studies covered both the adoption and use of energy technology. We labeled the first study “Adoption” since it describes a generic energy technology (the technology was not defined), and habit formation was seen as an influencing factor in investment behavior. The second study was labeled as “Use” since it studied energy-efficient heating behavior emerging from processes like technology diffusion.

#### 3.2.2 Categorizing behavioral factors and behavioral theories

To give insight into the fit between behavioral factors and theories (BFTs) and the simulated behaviors in the ABMs, we first assessed which specific BFTs were formalized in the ABMs.

We categorized the factors leading to behavior into three categories: “individual,” “social,” and “external” factors. Individual factors contain psychological factors (e.g., attitudes toward new technology or trust in energy companies) and demographics (e.g., age, family size, employment status). Social factors (e.g., network density, norms) are external drivers of behavior, but are separated as a single category since ABMs almost always contain a social component. The external factor category includes technological, economic, and environmental factors (e.g., building characteristics, price of technology, or weather conditions).

A distinction is made between the formalization of behavioral theories and the formalization of behavioral factors. This decision was empirically driven, as only a few studies formalized a complete theory, and many studies applied more than one behavioral factor.

Only actual formalizations were assessed. By this, we mean that if a behavioral factor was explained in the introduction as an important precursor to the behavior in question, it was not scored when it was not formalized. In the same line of reasoning, if a theory was explained to be relevant for the behavior in question, but not all parts of the theory were formalized in the model, it was not labeled as a theory but as separate behavioral factors. For example, if the Theory of Planned Behaviour (TPB, Ajzen, 1991) was mentioned as important, but “attitude” was the only theory concept formalized, “attitude” was coded as an individual behavioral factor. The label “theory” was not attributed to the paper.

This labeling and categorization of behavioral factors and theories provided us with an unbiased view of emerging themes in our data, whilst still being able to analyze and discuss the results within a behavioral science framework.

#### 3.2.3 Reasoning for applying behavioral factors and theories

To assess the clarity and validity of the reasoning for applying a behavioral factor or theory, we assessed (1) which arguments authors gave for choosing a behavioral factor or theory, and (2) if a behavioral analysis supported the choice of a behavioral factor or theory. The notions of “clear” and “valid” reasoning are separated in this analysis because an argument can be clear but not valid, and vice versa. “Clear” reasoning is attributed when arguments for the choice of a behavioral factor or theory are explicitly stated and can be retraced (regardless of the content of the argument). “Valid” reasoning for applying behavioral factors or theories is attributed when the argument is specific to the modeled household energy behavior or is backed up by a behavioral analysis. We elaborate on this below.

#### 3.2.4 Arguments

We distinguish between three categories of supporting arguments to explain the choice of behavioral factor or theory that leads to household energy behavior: (1) no arguments, (2) general arguments, and (3) specific arguments. The “no arguments” category holds the studies in which authors did not give an argument, for example, by merely stating which BFT was formalized. With “general arguments,” we mean the argument is present but behavior-unspecific. By not being behavior-specific, we mean that the argument takes a different energy behavior than the modeled energy behavior as a reference, for example, when the adoption of general energy technology is taken as a reference when modeling heat pump adoption or by stating that the Theory of Planned Behaviour (Ajzen, 1991) is a widely used social science theory. This category is also scored when studies state they extended or built on the work of others without being specific about why this other research chose behavioral factors and theories. The “specific argument” category was scored when an intentional focus on the applicability of the factor or theory to the modeled behavior was suggested. It was scored when (a) authors stated the behavioral factor or theory was underrepresented or understudied in ABMs, and/or (b) when authors gave reasons for the choice of a behavioral factor or theory that took the specific modeled behavior as a reference point (whether backed up by a behavioral analysis or not). The first criterion was chosen under the assumption that when authors state a behavioral factor or theory is underrepresented or understudied in ABMs, they have considered its relevance to the specific behavior being modeled or aim to test the theory in that specific context.

#### 3.2.5 Behavioral analyses

We labeled the presence of a behavioral analysis into four categories: (1) fully present, (2) mostly present, (3) partially present, and (4) not present. A behavioral analysis was scored as “fully present” when it covered a description or analysis of all four elements of a behavioral analysis as described in chapter 2: type of behavior or behavior change addressed in the model; individual and external factors influencing the modeled behavior; existing behavioral theories matching the household energy behavior addressed in the model; and reflection on data-gathering requirements for empirical grounding of the model.

Since none of the studies in our data set performed a full behavioral analysis, a BA was scored as “mostly present” when researchers performed at least step 2 and mentioned previous research on individual and external factors influencing the *specific* modeled household energy behavior. A behavioral analysis was scored as “partially present” if the authors did perform steps two and/or three but when this research was not specific to the household energy behavior addressed in the model or when, for example, one or a few individual constructs were researched in detail while other influencing factors were not discussed. Finally, behavioral analysis was scored as “not present” in cases where no behavioral analysis was performed at all. Table 1 illustrates the categories as scored in the dataset.

**Table 1:** Categories of behavioral analysis (BA) scored in the data set.

**Category labels**	**Description**
BA is fully present	BA steps 1 to 4 are included
BA is mostly present	At least BA step 2 is included, and the analysis is specific to the behavior in question
BA is partially present	BA step 2 and/or 3 are included, but the analysis is not specific to the behavior in question
BA is not present	BA steps 1 to 4 are not included

The steps in the second column refer to the four-step approach mentioned in Chapter 2 and Figure 1.

## 4 Results

In the following three sections, we first discuss which household energy behaviors and corresponding BFTs are studied in ABMs in Section 4.1. We then lay out the results of our analysis on the reasoning behind the choice of BFTs in Section 4.2. Both Sections 4.1, 4.2 include a comparison between Adoption- and Use-ABMs. We then analyze the (improved) fit when conducting a behavioral analysis in Section 4.3 and summarize the main results in Section 4.4.

### 4.1 Simulated energy behaviors and their formalized influencing factors

To answer our first research question (*RQ1: Which behaviors, behavioral factors and theories (BFTs) are studied?*), we took stock of the simulated household energy behaviors and the formalized factors influencing those behaviors.

#### 4.1.1 Energy behaviors

Our review contains the analysis of 71 studies published between 2009 and 2024. Roughly two-thirds of the articles in our review studied household adoption of energy technology (*n* = 49; 69%). The rest of the articles studied energy technology use (*n* = 22; 31%).

Table 2 displays the behaviors modeled in the “Adoption-ABM” category. The diffusion of rooftop solar panels (*n* = 18) and full electric or plug-in electric vehicles (*n* = 8) are modeled most in this adoption category. Furthermore, doing a retrofit and/or insulation of the house was modeled (*n* = 6), just like the adoption of full electric heat pumps (*n* = 3) and energy feedback devices (*n* = 3). A few ABMs did not define the technology (*n* = 3). The adoption of energy-efficient lighting (*n* = 2), the adoption of woodstoves (*n* = 2), agreeing with a connection to district heating (*n* = 2), the adoption of natural gas vehicles (*n* = 1), and the adoption of a dynamic tariff scheme (*n* = 1) closes the list.

**Table 2:** Behaviors in Adoption- (left) and Use- ABMs (right).

**Adoption-ABMs**	**Frequency**	**Use-ABMs**	**Frequency**
Buying PV	18	Energy consumption or reduction	9
Buying EV	8	*Energy consumption behavior*	*3*
*Full electric*	*6*	*Energy saving behavior*	*2*
*Plug-in hybrid*	*1*	*Adjust to heat or cold*	*1*
*Undefined*	*1*	*Ventilating rooms*	*1*
Doing a retrofit	6	*Use of heat pump*	*1*
*Isolation*	*4*	*Rebound buying EE lightning*	*1*
*Isolation + retrofit*	*2*	Load shifting	7
Buying full electric HP	3	*Load profile shift in general*	*5*
Buying feedback devices	3	*Off-peak EV charging*	*2*
*In-home displays*	*2*	Sharing energy use	6
*Adopting smart meter*	*1*	*Shared PV*	*4*
Tech. adoption, generally	3	*Shared EV + battery system*	*1*
Buying light bulbs	2	*Group decisions in HoA*	*1*
Buying wood stoves	2		
Connecting to district heating	2		
Buying natural gas vehicles	1		
Adopting a dynamic tariff	1		

PV, photovoltaic (solar panels); EV, electric vehicle; HP, heat pump; EE, energy efficient; HoA, homeowners association.

Subsequently, Table 2 displays the behaviors modeled in the “Use-ABM” category. Reducing household energy demand was modeled most (*n* = 9). It holds energy consumption or energy saving (*n* = 5), adjusting to heat or cold (*n* = 1), ventilating rooms (*n* = 1), use of heat pump (*n* = 1), and the rebound effect of energy-efficient lighting (*n* = 1, the rebound effect describes an increase in energy consumption after having made an energy efficiency decision). Furthermore, household load shifting decisions were modeled in seven studies, mostly computed for a shift in profile for the total energy consumption of a household (*n* = 5), but also for shifting electric vehicle charging to other parts of the day (*n* = 2). Shared use of energy systems within groups of households (*n* = 6) closes the list, including sharing one source of solar energy (*n* = 4) and sharing electric vehicles and battery systems (*n* = 1). One model treated a homeowners' association's decision to renovate.

#### 4.1.2 Behavioral factors and theories

**Figure 3:**
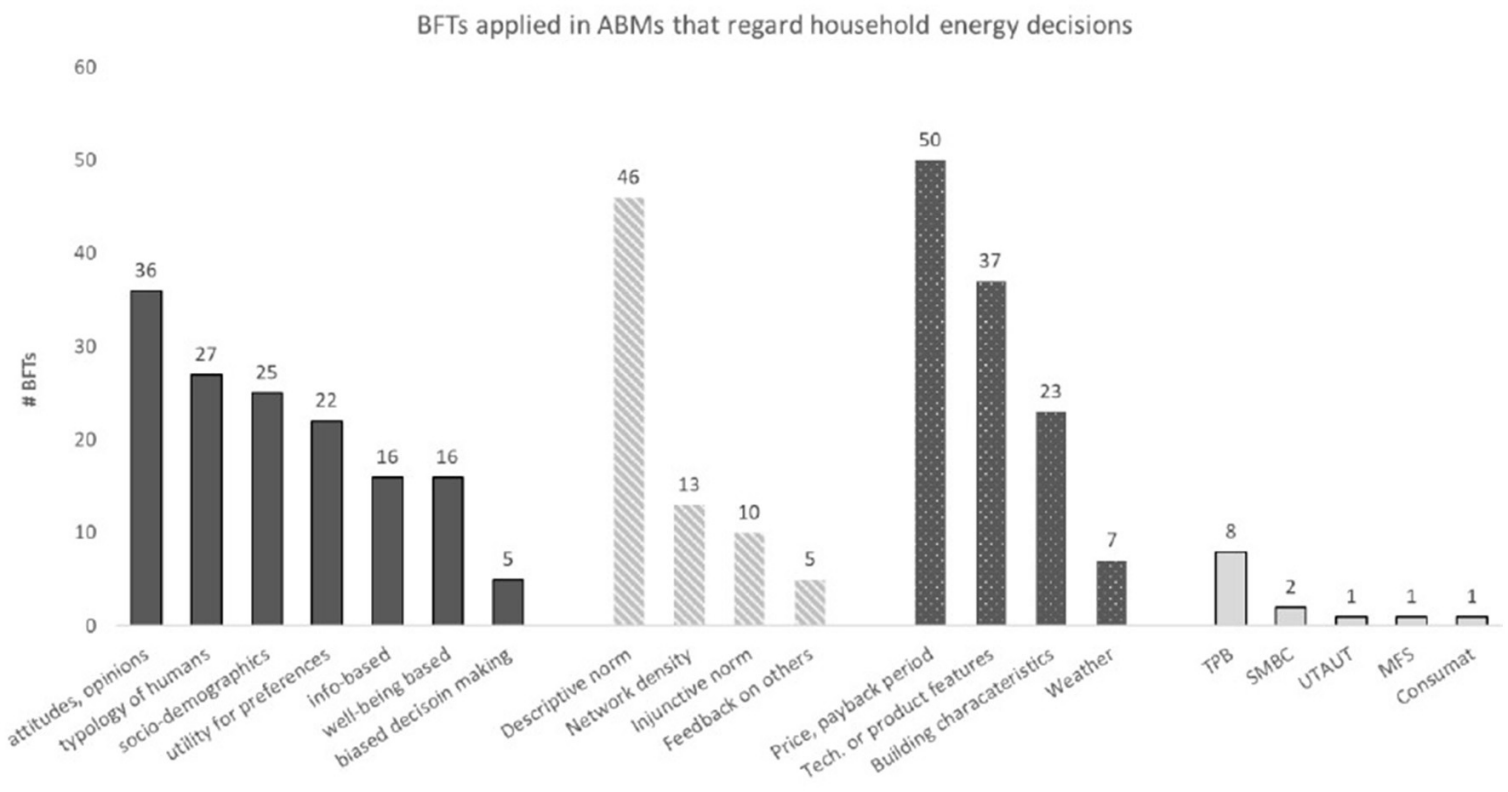
Prevalence of individual **(left)**, social **(mid)**, external **(right)** behavioral factors, and behavioral theories (most right) in ABMs that regard household energy behavior. TPB, Theory of Planned Behavior; SMBC, Stage Model of self-regulated Behavioral Change; UTAUT, Unified Theory of Acceptance and Use of Technology; MFS, Model of Frame Selection.

Because most studies formalize more than one behavioral factor or a combination of factors and theories, we report here the number of instances (*n* = 337) that a factor or a theory (*n* = 13) is formalized in the data set. The results are split up into behavioral factors and behavioral theories. As the Methods section mentions, behavioral factors are categorized as individual, social, and external factors influencing behavior. A broad spectrum of applied behavioral factors within each category was found (see Figure 3). The types of factors and theories that are formalized are described below.

#### 4.1.3 Formalized individual factors

Of all factors, individual factors were formalized the most (*n* = 147; 43%). Within the category of individual factors, seven types are found. First, they include studies (*n* = 36) that formalize perceptions, opinions, and attitudes (e.g., Jensen et al., 2016; Palmer et al., 2015), including past behavior or past attitudes (e.g., Silva and Krause, 2016; Wang et al., 2018) and environmental concern (e.g., van der Kam et al., 2019; Wolf et al., 2015). Second, studies formalized typologies of humans based on preferences (*n* = 27). These are either randomly assigned or empirically driven clearly stated divisions into a few groups of agents, including preferences based on values about well–being (e.g., Yue et al., 2020), saving money (e.g., Faber et al., 2010) or saving the environment (e.g., McCoy and Lyons, 2014), big five personality traits (e.g. Shen et al, 2021), post-materialistic or conservatives worldviews (e.g., Jensen et al., 2015), satisfying or maximizing types of agents (e.g., Wolf et al., 2015) and types of agents based on Rogers diffusion of innovation theory (e.g., Adeptu et al., 2018; Alyousef et al., 2017). Third, socio-demographics were formalized (*n* = 25 studies), including age, gender, income, family structure, employment status, etc. (e.g., Mueller and de Haan, 2009; Noori and Sun, 2018; Tatari, 2016; Tian et al., 2021). Fourth, studies included utility or weighing factors for preferences (*n* = 22), either randomly assigned (e.g., Kotthoff and Hamacher, 2022; Zhang and Han, 2024) or empirically driven (e.g., Derkenbaeva et al., 2023; Schiera et al., 2019). Fifth, information-based factors (*n* = 16 studies) are formalized, including the source of information (Chappin and Blomme, 2022; Nurwidiana et al., 2021), website info converted to knowledge and/or memory (e.g., Danielis et al., 2023), checking smart meter regularly (e.g., Weron et al., 2018), receiving feedback on own consumption (e.g., Chappin and Blomme, 2022). Sixth, wellbeing-based factors are formalized in *n* = 16 studies. These include emotions like range anxiety (e.g., van der Kam et al., 2019), spiritual satisfaction (e.g., Yue et al., 2020), comfort (e.g., de Wildt et al., 2021; Meles and Ryan, 2022) and hassle or perceived hassle (e.g., Snape et al., 2015). Seventh, proneness to biased decision-making was formalized in 5 studies, including inertia (e.g., Stavrakas et al., 2019), loss aversion (e.g., Chen et al., 2023), and information bias (e.g., Ebrie and Kim, 2023).

#### 4.1.4 Formalized social factors

Social factors are formalized in 21% of the cases (*n* = 74). Within the category of social factors, four types are found in the data set. First, studies include descriptive norms (*n* = 46), meaning agents copy the behavior they see others doing, either randomly assigned (e.g., Chen et al., 2020) or empirically driven (e.g., Friege, 2016; Opiyo, 2019), mentioned as the number of “friends” (e.g., Alyousef et al., 2017) or “neighbors” (e.g., Egner and Klöckner, 2022; Kowalska-Pyzalska et al., 2014) from which the behavior is copied, or as the contagion of behavior (e.g., Moglia et al., 2022), and as social learning (e.g., Golmaryami et al., 2024; Khansari and Hewitt, 2020). Second, network density or nodes are formalized in 13 studies (e.g., Alderete Peralta et al., 2022). Third, studies also include injunctive norms (*n* = 13). An injunctive norm is activated when people act because they perceive that others think it is right. These mention pro-social behavior or social value orientation (e.g., Silva and Krause, 2016), social comparison or acceptance (e.g., de Wildt et al., 2021), peer pressure or sensitivity to the behavior of peers (e.g., Walzberg et al., 2019) or word of mouth with a suggestion to adopt (e.g., Danielis et al., 2023). Fourth, feedback provisions on relevant others are formalized (*n* = 5 studies) when agents react to injunctive and/or descriptive feedback on what others do (e.g., Chen and Wei, 2012).

#### 4.1.5 Formalized external factors

External factors are formalized in 36% of the cases (*n* = 117). Within the category of external factors, four types are found. First, studies include economic features (*n* = 50) like the price of technology or service (e.g., Hicks and Theis, 2014; Maqbool et al., 2019; Wang et al., 2018;), payback periods (e.g., Palmer et al., 2015; Stavrakas et al., 2019) or assumptions of adoption-increase based on recession or inflation arguments (e.g., Jagadish et al., 2019). Second, technological or product features are formalized in 37 studies; examples include ownership of solar panels, electric vehicle, heat pump, etc. (e.g., Madler et al., 2023), state of charge for electric vehicles (e.g., Williams et al., 2024), and the presence of an on/off switch button on a heat pump (Chen et al., 2020). Third, building characteristics are mentioned in 23 studies, including the type of building (e.g., Mussawar et al., 2023), insulation value of the house (e.g., Nava-Guerrero, 2022), and floor area (e.g., Sun et al., 2018). Fourth, weather conditions are formalized (*n* = 7 studies), including household consumption data that are corrected for outside temperature (e.g., Khansari and Hewitt, 2020) or electricity demand or the range of electric vehicles depending on the season (e.g., Madler et al., 2023).

#### 4.1.6 Formalized behavioral theories and frameworks

No more than 13 studies (18% of the total data set) formalized one or two of five different behavioral theories or frameworks, see Figure 3. Appendix B describes the five applied behavioral theories.

The Theory of Planned Behavior (TPB, Ajzen, 1991) was applied most in eight studies (Caprioli et al., 2020; Jensen et al., 2016; Lee and Malwaki, 2014; Meles and Ryan, 2022; Muelder and Filatova, 2018; Rai and Robinson, 2015; Schiera et al., 2019; Sopha et al., 2011), followed by Bamberg (2013)'s Stages Model of self-regulated Behavior Change (SMBC) in two studies (Lee and Malwaki, 2014; Weron et al., 2018). Three behavioral theories and frameworks were formalized once: the Unified Theory of Acceptance and Use of Technology (UTAUT, Venkatesh et al., 2003) is applied in Nurwidiana et al. (2021); the Model of Frame Selection (MFS, Esser and Kroneberg, 2015) is applied in Hoffman et al. (2020), and the Consumat framework (Jager et al., 2000) is applied in Derkenbaeva et al. (2023). For which type of behavior and to what level of detail the different theories are applied is broken down below.

The studies that formalized the TPB, modeled the adoption of rooftop solar panels (*n* = 4), heat pumps (*n* = 1), woodstoves (*n* = 1), and CO_2_ meters (*n* = 1). In one paper, the TPB was applied to model energy-saving behaviors (adjusting clothing and fan use). In all studies, the TPB was formalized at the attitude, subjective norm, and perceived behavioral control level. The studies did not systematically break down the factors of the theory that shape the three main concepts of the TPB (see Appendix B).

The studies that formalized the SMBC modeled energy-saving behaviors (adjusting clothing and fan use) and the impact of training programs on the diffusion of web-based smart metering platforms. Both studies formalized the level of the different stages and (some, not all) different types of intentions in this theory. Like the studies that applied the TPB, these studies did not systematically break down all the concepts in the theory (see Appendix B). In this case, the factors that shape the four stages and intentions of the SMBC were not formalized.

The study that formalized the UTAUT simulated households' intention to adopt solar panels. The theory was formalized almost completely: the concepts of “experience with technology” and “voluntariness of use” were not included. Regarding the other two theories, one study formalized the MFS, and one formalized the Consumat framework. The first simulated the adjustment of energy consumption of households in a grid after receiving different kinds of information; the latter modeled the adoption of energy-efficient retrofitting of households. For both, all parts of the theory and the framework were formalized (see Appendix B).

#### 4.1.7 Difference between Adoption- and Use-ABMs in applying BFTs

**Figure 4:**
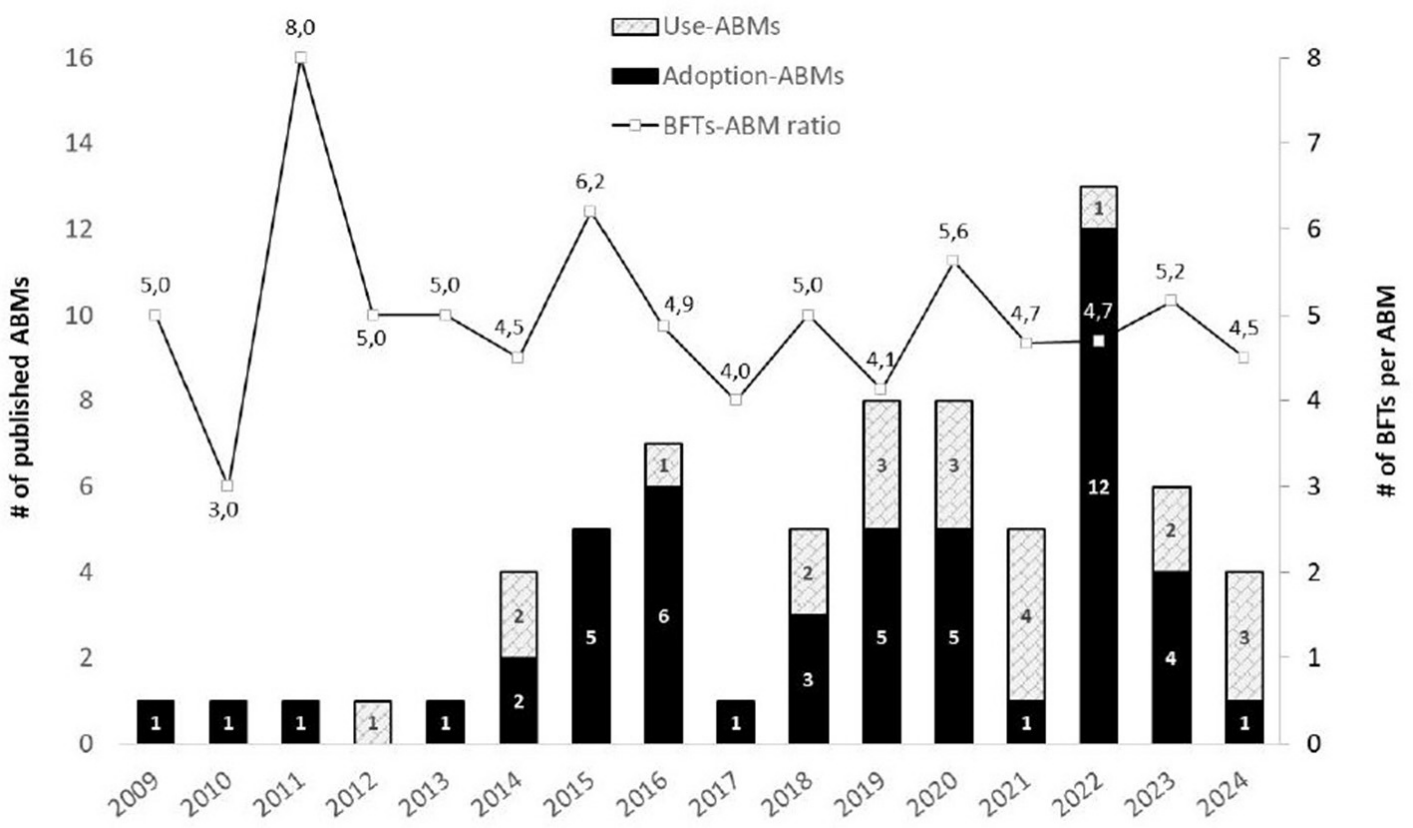
Year of publication for the 49 Adoption-ABMs and 22 Use-ABMs in our data set (bars, left y-axis), and the number of behavioral factors and theories (BFTs) per ABM (line, right y-axis). Publications for 2024 were included up until February 2024. We checked the dip in 2017 for a methodological flaw but could not find it. The dip and rise of publications in 2021 and 2022 could be due to the COVID period. Generally, we see an increase in the number of publications per year and a more or less steady ratio of BFTs per ABM.

First, we assessed the increase in the publications of ABMs for both Adoption and Use-ABMs over time. We report a steady increase in the number of total publications per year: more than 50% of the articles were published in 2019 or later, revealing the increasing popularity of ABM to study the role of households in the energy transition. Adoption-ABMs have a longer track record than Use-ABMs: Adoption-ABMs first saw light in 2014, compared to 2009 for Use-ABMs. Also, the mean amount of BFTs formalized in ABMs does not increase over time (see Figure 4).

We also assessed the difference in the application of BFTs for Adoption and Use-ABMs. Use-ABMs formalize fewer behavioral factors (BFs) than Adoption-ABMs. First, for behavioral factors, the ratio per ABM is 4.1 for Use-ABM and 5.0 for Adoption-ABMs. Second, for behavioral theories (BTs), the ratio is the same for Use-ABM and Adoption-ABMs (both 0.18) (Table 3).

**Table 3:** Ratio of behavioral factors (BFs) and behavioral theories (BTs) per ABM.

**Application**	**Adoption-ABMs**	**Use-ABMs**
#ABMs	49	22
# BFs	247	90
# BTs	9	4
Ratio BF/ABM	5.0	4.1
Ratio BT/ABM	0.18	0.18

Results are split up for Adoption- and Use-ABMs.

Additionally, we assessed whether the application of the type of BFTs differs between adoption- and use-ABMs. For behavioral factors, only minor differences are found. Use-ABMs formalize individual factors in 44% of the cases (compared to 36% for Adoption-ABMs), social factors are formalized in equal amounts for Adoption- and Use-ABMs (both 22%), and external factors are formalized in 32% of the cases for Use-ABMs (compared to 40% for Adoption-ABMs). This finding is surprising, as we expected a difference due to the different nature of both behaviors.

In contrast, a notable difference was found in applying behavioral theories for adoption-ABMs and use-ABMs. Full theories are not applied much, but when applied, they are applied less in Use-ABMs (*n* = 3; 12%) than in Adoption-ABMs (*n* = 10; 21%). This difference is most prominent for the TPB; it is applied in one instance for Use-ABMs (Lee and Malwaki, 2014) and in seven instances for Adoption-ABMs. One could expect TPB application for adoption behaviors, as the TPB is a theory explaining reasoned actions. Other formalizations of full behavioral theories are scarce, as two other cases for Use-ABMs (SMBC, MFS) and three other instances for Adoption-ABMs (SMBC, UTAUT, Consumat) were found.

### 4.2 Reasoning for the choice of behavioral factors and theories

To answer our second research question (*RQ2: Is the reasoning for the choice of BFTs stated clearly and does a behavioral analysis back up the reasoning?*), we took stock of the supporting reasons for the choice of BFTs provided by authors by assessing (a) which arguments were given by authors for the choice of a BFT and (b) if a behavioral analysis supported the choice of a BFT.

#### 4.2.1 Clear arguments: is an argument provided for the choice of a BFT?

**Figure 5:**
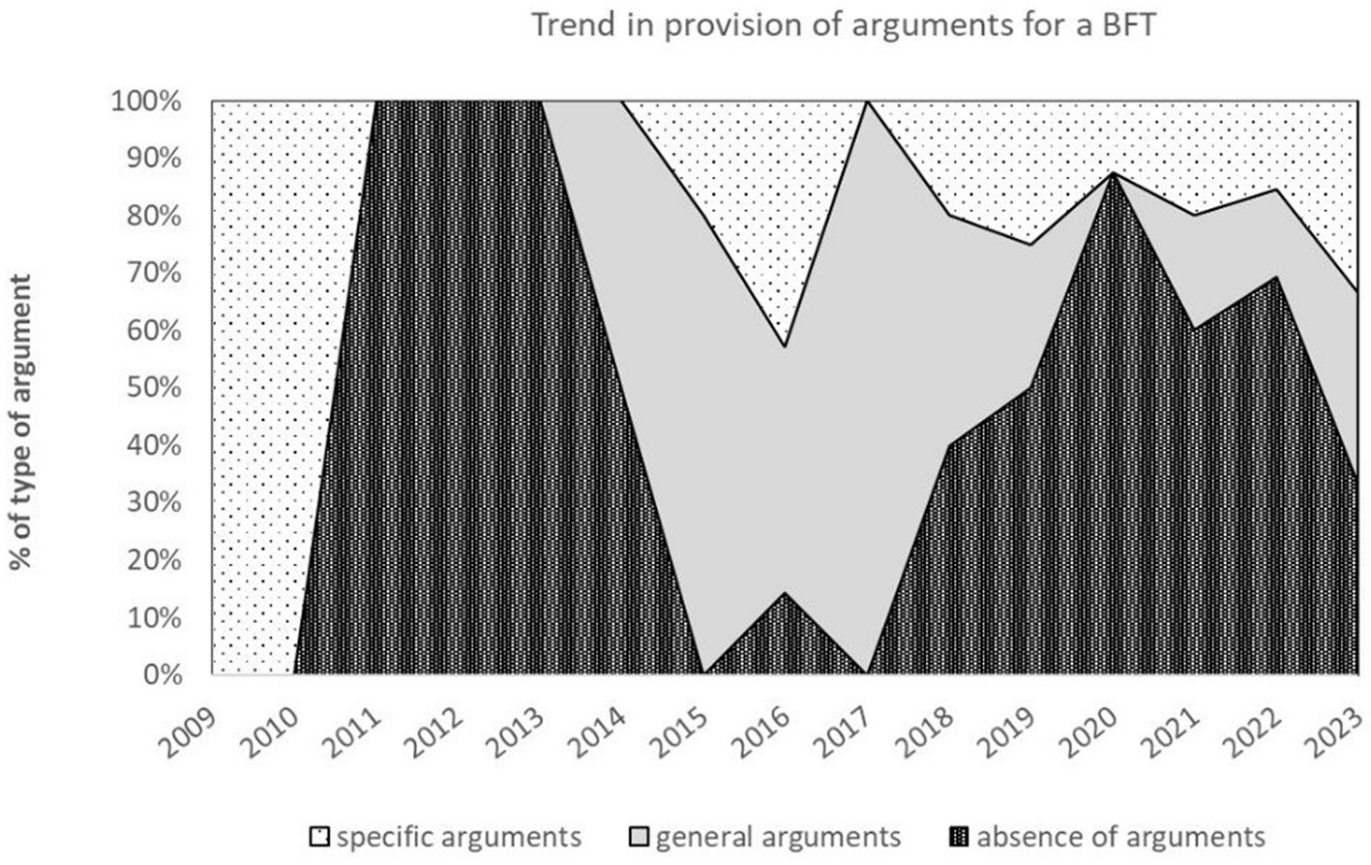
Area chart showing trends in the provision of arguments for a BFT from 2009 to 2023. There is an absence in trend for clear reasoning for the choice of behavioral reasoning for the choice of behavioural factors and theories. Absence in trend for clear reasoning for the choice of behavioral factors and theories. The mean 2-1-1 ratio of the absence of arguments, general arguments, and specific arguments fluctuates over time.

Almost half the studies (*n* = 35; 49%) did not state why they formalized a behavioral factor or theory. Slightly more than a quarter of studies (*n* = 19; 27%) gave a general argument (they stated that they copied the work of others or gave an argument based on a broader behavioral domain than the modeled household behavior). The remaining quarter (*n* = 17; 24%) of the articles gave a more specific argument: either they stated that the BFT was underrepresented in ABMs (*n* = 11, e.g., Neshat et al., 2023; Huang et al., 2022), or the argument covered that the BFT was specifically suited for the modeled household energy behavior because of reason x or y (*n* = 6, e.g., Faber et al., 2010; Zhang et al., 2016). Figure 5 depicts the general trend for providing arguments over time until 2023 (we excluded the year 2024 because our search didn't include the whole year, which skews the results). From 2015 on, specific arguments for BFTs are given for about 20% of the studies.

#### 4.2.2 Valid arguments: is the choice for a BFT backed up by a behavioral analysis?

**Figure 6:**
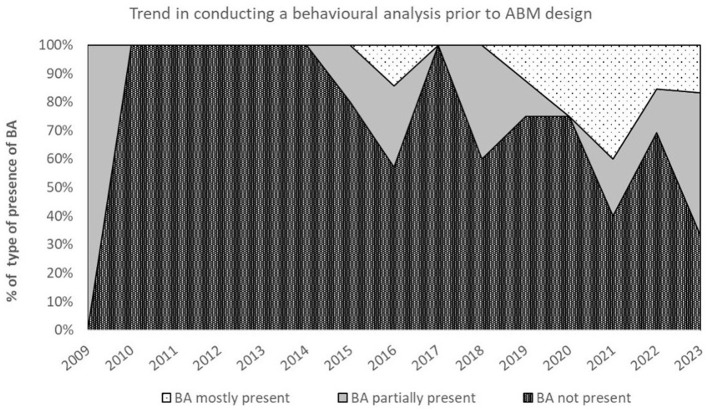
The trend in conducting a behavioral analysis prior to model design. The practice of behavioral analysis is an upcoming phenomenon for ABMs that simulate household energy decisions.

None of the studies in our data set conducted a full behavioral analysis as defined in the Methods section (“fully present”). Nevertheless, a behavioral analysis was “mostly” or “partially” present in roughly one-third of the studies (*n* = 24; 34%). Fifteen of these 24 studies conducted a partial behavioral analysis. In these studies, the behavioral analysis was not specific to the household energy behavior simulated in the model, or a few behavioral factors were researched in detail while other influencing factors were not discussed (e.g., Moglia et al., 2022; Yue et al., 2020). Nine of these 24 studies conducted a behavioral analysis that was scored as “mostly present” (Derkenbaeva et al., 2023; Golmaryami et al., 2024; Mueller and de Haan, 2009; Neshat et al., 2023; Palmer et al., 2015; van der Kam et al., 2019; Weron et al., 2018; Zhang et al., 2016; Zhang and Han, 2024). In these cases, the behavioral analysis was specific to the modeled behavior and considered previous research on possible individual and external factors influencing the modeled behavior. Figure 6 depicts the trend for conducting (parts of) a behavioral analysis up until 2023. The method seems to be an upcoming practice as the complete absence of a behavioral analysis seems to be declining.

#### 4.2.3 Difference between Adoption- and Use-ABMs in reasoning for BFTs

When Adoption- and Use-ABMs are compared for how behavioral factors and theories (BFTs) are reasoned for, we first find that the absence of arguments is more prominent in Use- than in Adoption-ABMs: 70% of the studies that modeled a Use-behavior did not report back on why they chose a BFT, compared to 41% for Adoption-ABMs. Specific arguments are given equally for Adoption- and Use-ABMs (24% and 22%, respectively). Second, we find that the method of (applying parts of a) behavioral analysis is slightly more practiced for Adoption-ABMs: 36% of the Adoption-ABMs “partially” or “mostly” perform a behavioral analysis, compared to 26% of the Use-ABMs.

### 4.3 Improvement in fit when a behavioral analysis is performed

To answer our third research question (*RQ3: Does the fit of BFTs with the modeled behavior improve when a behavioral analysis informs the choice of these BFTs?*), we assessed the difference between Adoption- and Use-ABMs for whether or not the amount and the type of formalized BFTs changes when a behavioral analysis is done.

First, the number of applied behavioral factors (BFs) per ABM increases slightly when a behavioral analysis is conducted, for both Adoption- and Use-ABMs. Additionally, in Adoption-ABMs, the number of behavioral theories (BTs) incorporated tends to rise when a behavioral analysis is performed. Interestingly, in contrast, an opposite effect is observed in Use-ABMs: no behavioral theories are formalized at all when a behavioral analysis is carried out. See Table 4 for an overview.

**Table 4:** Small increase in ratio of behavioral factors (BFs) and behavioral theories (BTs) per ABM when a BA when a is mostly performed (BA+) compared to when a BA is not or partially performed (BA–).

**Application**	**Adoption-ABMs**		**Use-ABMs**	
	**BA (–)**	**BA (+)**	**BA (–)**	**BA (+)**
#ABMs	38	11	18	4
# BFs	188	59	72	18
# BTs	6	3	4	0
Ratio BF/ABM	4.9	5.4	4.0	4.5
Ratio BT/ABM	0.16	0.27	0.22	0

Results are split up for Adoption- and Use-ABMs. Note that for Use-ABMs, no behavioral theories are implemented when a BA is conducted.

**Figure 7:**
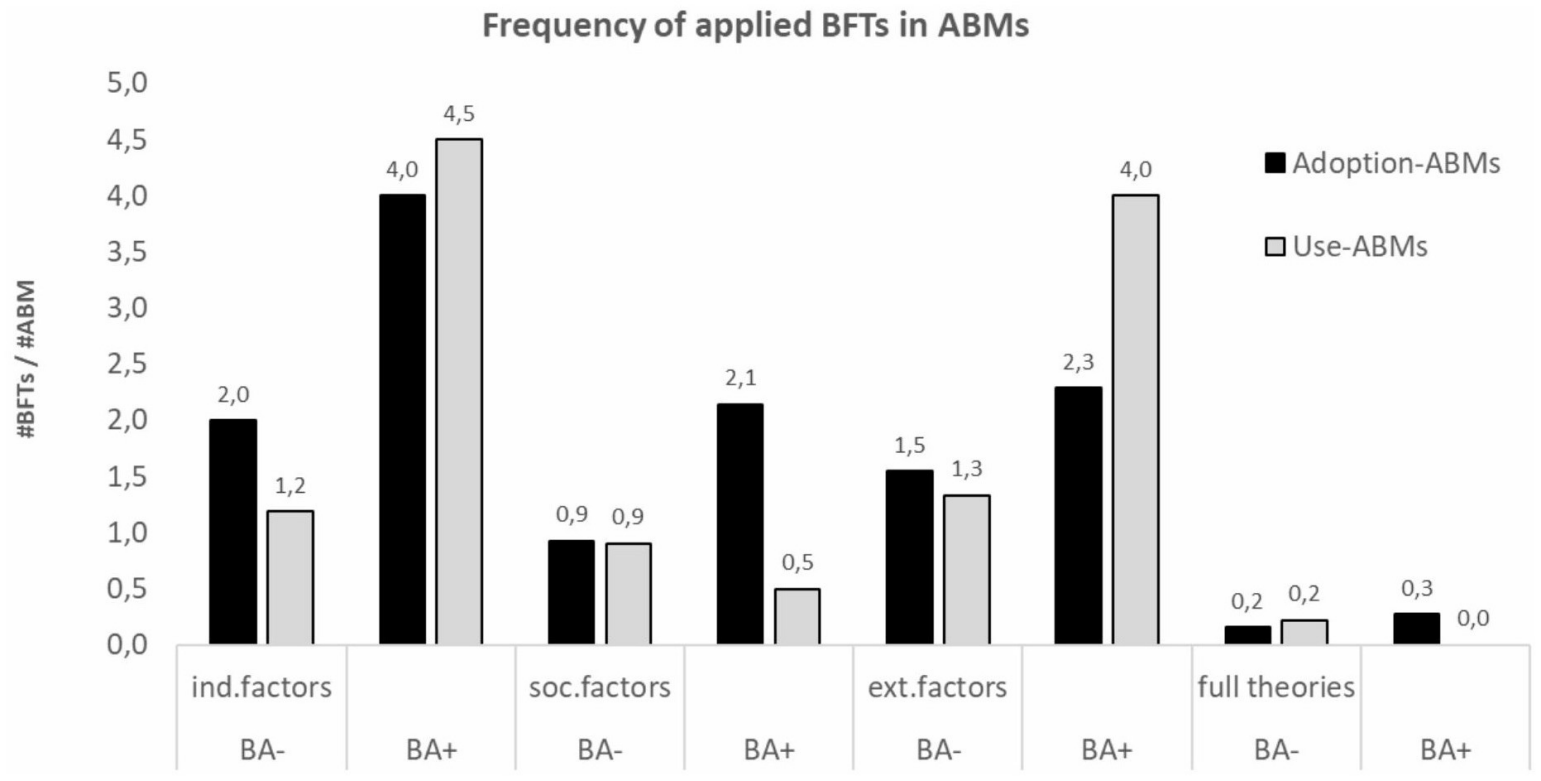
The difference in frequency of applied BFTs between Use- and Adoption-ABMs when a BA is performed (e.g., individual factors are applied more in Adoption-ABMs when a BA is present, the factor-ratio increases from 2.0 to 4.0). BA, means that the BA is not or partially present; BA+, means it is mostly present.

Second, the change in type of applied BFTs when a behavioral analysis is conducted, differs between Adoption and Use-ABMs. Adoption-ABMs formalize more of each type (behavioral factors and theories) of BFTs when a behavioral analysis is (mostly) present. In contrast, Use-ABMs formalize more individual and external factors, but less social factors and behavioral theories when a behavioral analysis is (mostly) present (Figure 7).

### 4.4 Synthesizing main results

In this chapter, three main results from the findings in Sections 4.1–4.3 are distilled. First, we conclude that a modeler's choice for an agent's decision style is (at least partly) driven by how suitable behavioral factors and theories are for implementation in an ABM, rather than by their relevance for accurately representing the behavior in question. This conclusion is based on the following three research findings. First, when a behavioral theory is employed, it is primarily the TPB, which has clear building blocks that are relatively easy to implement in an ABM. However, the theory is not fit to explain every household energy behavior equally well, and it is important to recognize that there are many more behavioral theories that offer suitable explanations of behavior. Second, if a social factor is formalized, it is primarily the descriptive norm (the rules that a person believes they should follow because they perceive that most others follow that rule). The prevalence of this factor could very well be related to the suitability of ABMs to simulate the imitation of a certain number of “neighbors” or “friends.” Third, many studies do not provide a clear or valid reasoning for choosing a BFT. Half of the studies offer no argument, and only one-third of the studies conducted a partial behavioral analysis. While this does not confirm that “suitability for implementation” is the main reason for applying behavioral factors or theories (BFTs), the lack of retraceable argumentation does suggest that the fit between BFTs and behavior is not the main driver of a modeler's decision.

Second, the findings indicate that the fit between BFTs and simulated behavior improves when conducting a behavioral analysis. This conclusion is based on two results. First, when a more comprehensive behavioral analysis is conducted, the number of behavioral factors (BFs) per ABM increases for both Adoption- and Use-ABMs (see Table 3), indicating that such analysis gives a more detailed view of the factors influencing behavior. Second, when a behavioral analysis is conducted, Use-ABMs formalize less social and more external factors. We consider this shift as the first indication of an improved fit because (1) the Use-behaviors in our data set are mostly actions inside the house, unseen by others, which makes them less prone to social influence, and (2) Use-behaviors are habitual in nature and therefore generally more prone to heuristic decision-making (individual factors) and environmental cues (external factors) than Adoption-behaviors. Caution in this line of reasoning is required: determinants of behavior are not only dictated by the reflective or habitual nature of behavior, they also depend for a great deal on the type of behavior change (e.g., stop old behavior or learn completely new behavior) and on which behavior is studied precisely (e.g., the adoption of heat pumps by households is influenced by other factors than the adoption of solar panels).

A cautionary note is necessary for the results mentioned above, as some of the reported differences between Adoption- and Use-ABMs are based on cells that contain low numbers. This applies especially to two groups: the amount of ABMs with a behavioral analysis “mostly present” (Use-ABMs, *n* = 4; Adoption-ABMs, *n* = 11), and the amount of ABMs that formalized a complete behavioral theory (Use-ABMs, *n* = 4; Adoption-ABMs, *n* = 9). Please note that reported differences between these groups are merely indicative and that observed trends could shift with a larger sample.

Third, simulating household energy behaviors that require less deliberative thinking (the “Use” behaviors, e.g., ventilating rooms, shifting energy practices to other parts of the day, or using energy from a shared source), is a less developed field than the simulation of households adopting an energy technology. The following three research findings corroborate this result. First and most importantly, twice as many Adoption-ABMs as Use-ABMs are found. Second, the development of Use-ABMs started later than the development of Adoption-ABMs. Although “younger” does not necessarily mean “less developed,” it does give an indication. Third, although the number of implemented theories decreases in both Adoption- and Use-ABMs when a behavioral analysis is conducted, in the case of Use-ABMs, it resulted in a complete absence of behavioral theory implementation. Together, these findings point to a research gap and highlight the need for further development and theoretical grounding of Use-ABMs in the energy behavior domain.

## 5 Discussion

### 5.1 Methodological implications

This section interprets the main findings and situates them in existing literature to provide context. The finding that suitability for implementation in ABM is an important driving force for choosing a BFT aligns with existing literature, which has shown that decision rules in ABMs are simplified and not grounded in well-established behavioral theories (e.g., Groeneveld et al., 2017; Parker et al., 2002). More recently, Senkpiel et al. (2020) reviewed the consideration of behavioral and psychological aspects in energy system models and concluded that models primarily focus on small areas of social science, such as acceptance. Furthermore, Mehdizadeh et al. (2022) reviewed the application of ABM in mobility transition studies and found that half of the reviewed papers apply a “heuristic algorithm:” decision rules that are simplified and not derived from well-established behavioral theories. Similarly, in a review study about the application of ABM to understand pro-environmental behavior, Ribeiro-Rodrigues and Bortoleto (2024) concluded that the TPB and social norms were used most prevalently to simulate agent decision-making. They highlight that most studies justified the choice of TPB “due to its popularity” and that ABMs focus on social norms mostly, emphasizing a narrow view of psychological influences on behavior.

A deeper integration of social science literature into ABMs can be facilitated by clearly reporting the rationale behind the choice of behavioral determinants. This allows researchers to build on existing knowledge by understanding why certain BFTs were selected over others (Wijermans et al., 2023). Moreover, it creates room to discuss conflicting ideas about the fit of determinants with behavior. This, in turn, enhances the incorporation of relevant behavioral insights in future models. We propose that arguments should at least (a) hold the purpose of the model, (b) be specific to the behavior in question, and (c) explain if and why the work of others was used to build on.

To further promote the behavioral realism of agent decision-making in a model, the findings suggest that a behavioral analysis can be of use. While the literature describing how to integrate behavioral insights in ABM design remains relatively underdeveloped, the first approaches are being published. For instance, Wijermans et al. (2023) introduce five behavioral frameworks that assist agent-based modelers in navigating the multidisciplinary boundaries inherent to their work. Furthermore, they propose a set of guiding questions that can help to critically examine how behavioral factors align with the behaviors under investigation.

Effectively incorporating behavioral insights into ABMs requires balancing the depth of behavioral understanding, on the one hand, with the need for model simplicity on the other. Because ABMs rely heavily on behavioral rules, a structured examination of the behavior studied in the model is essential for integrating relevant behavioral insights. At the same time, adding complexity without justification can lead to overfitting, reduced interpretability, and increased computational costs, making models harder to validate and apply effectively. Therefore, we argue not for indiscriminate increases in detail, but for maintaining a balance between behavioral realism and model simplicity. Conducting a behavioral analysis before model development can help with this, by prioritizing the most influential behavioral factors, ensuring that only determinants with significant explanatory power are included.

The finding that simulating behavior with a more habitual nature (the “Use” behaviors) is a less developed field than simulating behaviors with a more reflective nature (the “Adoption” behaviors), is also an apparent issue in social science literature. For example, it has been argued that psychological interventions typically fail to address the habitual nature of behavior (e.g., Cash et al., 2023; Barr et al., 2005), and that there is a need for climate policies that stimulate consistent environmental behavior beyond short-term voluntary actions (e.g., Dubois et al., 2019). By adopting structured behavioral analyses, future ABM studies can help fill this gap, advancing both modeling practice and theory development in the social sciences by deepening our understanding of how household energy habits form and change.

### 5.2 Directions for future research

The proposed behavioral analysis approach, which advocates for systematically examining the factors underlying simulated behavior in agent-based models, marks a pioneering effort in its field, paving the way for further development and improvement. Several significant opportunities arise from this initial effort.

First, more thorough research and interviews with model designers are needed to understand why modelers choose certain BFTs. The fact that nearly half of the studies under review did not report the reason for applying BFTs, does not mean that the reasoning behind the choice is absent. Reasons could, for example, include the purpose of the model (a perfectly valid reason to study a narrow set of BFTs). More thorough research into the topic would make reasoning more accessible to other researchers. A promising next step could be to systematically assess how behavioral analyses give input and meaning to the model purpose.

Second, future research could examine if data collection methods fit the data needed to ground the model empirically, and if this practice improves by performing a BA. Furthermore, it could be investigated if model outcomes improve by implementing different data types (e.g., survey data vs. data obtained from field trials), as the empirical grounding of the models in this study was not analyzed. For example, if the model included types of people (innovators, early adopters, early majority, late majority, laggards) based on Rogers (2003)'s Theory of Innovation Diffusion, it could be assessed if the distribution of agents in these groups were randomly assigned, followed a normal distribution or were based on empirical data.

Third and relatedly, as validation efforts hugely contribute to behavioral realism of ABMs, future methodologies could include (1) comparing model outputs with real-world behavioral data to determine whether ABMs that incorporate BA, more accurately predict observed behaviors, (2) cross-model comparison, where models with and without a BA are compared to see if those integrating a BA produce the more plausible or policy-relevant outcome, (3) expert assessments, where behavioral scientists assess models with and without BA on if the modeled behavioral mechanisms align with established behavioral theories and empirical evidence, and (4) taking a generative social science approach, where it could be examined whether ABMs with BA are more effective at “growing” observed social patterns from the bottom up.

Fourth, a promising next step could be to systematically assess how behavioral analyses improve the behavioral realism and quality of model results, given that this is done within specific domains of behaviors. Future research could develop criteria to evaluate whether and how behavioral analyses enhance model interpretability, predictive accuracy, and policy applicability. Assessing behavioral realism is incredibly difficult as concepts like “useful,” “valid,” or “relevant” model results are important to define precisely, and reaching consensus on these concepts for a whole field is impractical.

Last but not least, the definition of a behavioral analysis (BA) put forward in this review is a first effort to pinpoint the method. The four-step approach (see Figure 1) originates from the authors' training as social psychologists, their experience with research into energy behavior of households to improve policy design, and their experience with testing and applying behavioral insights into energy transition modeling. Although backed up by literature applying BA to policy design, and tailored to the practice of agent-based modeling, other researchers can make different decisions on whether or not to include elements of the BA and in which phase of model design a BA is most suited.

While we argue that a comprehensive behavioral analysis can enhance the realism and decision-support capabilities of ABMs to policymakers, integrating complex behavioral theories and data collection methods can be resource-intensive. As balancing rigor with feasibility is important in ABM design, future research could identify which aspects of BA provide the most value for a given modeling purpose. An alternative could also be to implement the levels of behaviorally aligned, informed, and tested insights in ABMs (see chapter 2, where we refer to Sousa Lourenço et al., 2016, who coined this concept for policy design), linked to model purpose. Furthermore, a behavioral analysis could or could not include expert elicitation on the topic (e.g., by means of interviewing behavioral domain experts), sensitivity analysis (testing the robustness of the psychological model), and/or empirical validation studies (including measurement validation and validation of the psychological model). We strongly encourage modelers to seek collaboration with behavioral scientists to grow interdisciplinary research on this topic.

## 6 Conclusion and recommendation

This review contains the analysis of a data set of 71 studies of agent-based simulated household behavior published between 2009 and 2024. The data set contains 337 instances of behavioral factors and 13 instances of behavioral theories that were formalized. We assessed if, and if so, which reasoning was provided for the choice of these behavioral factors and theories. Most importantly, we examined if a behavioral analysis before ABM design improved the uptake of behavioral insights in ABMs.

Our findings suggest that incorporating behavioral analysis enhances the behavioral realism of ABMs. More realistic models provide policymakers with useful insights, allowing for the design of energy policies more aligned with citizens' needs. These policies are more likely to gain public acceptance and encourage compliance, ultimately supporting a more effective energy transition that limits global temperature rise.

As none of the studies under review implement a full behavioral analysis, we deem integrating the practice of behavioral analysis in model design an important step forward. Broader familiarity with social science in shaping the choice for behavioral determinants in models is necessary for this step forward. That said, conducting a behavioral analysis is a challenging task that requires significant resources. Rigorously pinpointing the exact behavior of interest and identifying the factors that influence it, is complex, as the rapidly expanding psychological literature offers overwhelming empirical evidence on behavioral determinants. This vast array of information makes it difficult to discern what is most relevant. Furthermore, it can be hard for those without specialized training to recognize gaps, interpret findings accurately, and prioritize key insights in behavioral literature. This is why we deem interdisciplinary and team science crucial. Involving an interdisciplinary team, particularly with input from a behavioral psychologist, is key to navigating these complexities effectively. To overcome the challenges of interdisciplinary teamwork, it is very important to allow for sufficient time and resources for discussion (e.g., De Vries et al., 2021; Scholz et al., 2023; Trutnevyte et al., 2019; Valkengoed et al., 2024; Wijermans et al., 2022).

## Data Availability

The authors have provided an Excel file with publications as rows, and in columns the BFTs, type of behavior, level of behavior (Adoption or Use), levels of behavioral analysis, and levels of arguments. This Excel file can be found in the 4TU research data repository: https://doi.org/10.4121/42afa47f-d7f6-4860-bbfe-592a7682c3fa.
