# Supplementary material for: Bridging behavioral theory and household energy decisions: enhancing agent-based models with behavioral analysis
Source: Front Psychol. 2025 Jul 9;16:1568730. doi: 10.3389/fpsyg.2025.1568730 (PMC12284503; doi:10.3389/fpsyg.2025.1568730)
Supplement: Supplementary file 1 [file Supplementary_file_1.docx]

# Appendix A – Studies under review

Adepetu, A., Alyousef, A., Keshav, S., & Meer, H. de. (2018). Comparing solar photovoltaic and battery adoption in Ontario and Germany: an agent-based approach. *Energy Informatics*, *1*(1), 6. <https://doi.org/10.1186/s42162-018-0012-8>

Alderete Peralta, A., Balta-Ozkan, N., & Longhurst, P. (2022). Spatio-temporal modelling of solar photovoltaic adoption: An integrated neural networks and agent-based modelling approach. *Applied Energy*, *305*, 117949. <https://doi.org/10.1016/j.apenergy.2021.117949>

Alyousef, A., Adepetu, A., & de Meer, H. (2017). Analysis and model-based predictions of solar PV and battery adoption in Germany: an agent-based approach. Computer Science - Research and Development, 32(1), 211–223. <https://doi.org/10.1007/s00450-016-0304-9>

Boumaiza, A., Sanfilippo, A., & Mohandes, N. (2022). Modeling multi-criteria decision analysis in residential PV adoption. Energy Strategy Reviews, 39, 100789. <https://doi.org/10.1016/j.esr.2021.100789>

Byrka, K., Jȩdrzejewski, A., Sznajd-Weron, K., & Weron, R. (2016). Difficulty is critical: The importance of social factors in modeling diffusion of green products and practices. Renewable and Sustainable Energy Reviews, 62, 723–735. <https://doi.org/10.1016/j.rser.2016.04.063>

Cao, J., Choi, C. H., & Zhao, F. (2017). Agent-based modeling of the adoption of high-efficiency lighting in the residential sector. Sustainable Energy Technologies and Assessments, 19, 70–78. <https://doi.org/10.1016/j.seta.2016.12.003>

Caprioli, C., Bottero, M., & De Angelis, E. (2020). Supporting Policy Design for the Diffusion of Cleaner Technologies: A Spatial Empirical Agent-Based Model. <https://doi.org/10.3390/ijgi9100581>

Chappin, E. J. L., & Afman, M. R. (2013). An agent-based model of transitions in consumer lighting: Policy impacts from the E.U. phase-out of incandescents. Environmental Innovation and Societal Transitions, 7, 16–36. <https://doi.org/10.1016/j.eist.2012.11.005>

Chappin, E. J. L., Schleich, J., Guetlein, M.-C., Faure, C., & Bouwmans, I. (2022). Linking of a multi-country discrete choice experiment and an agent-based model to simulate the diffusion of smart thermostats. Technological Forecasting and Social Change, 180, 121682. <https://doi.org/10.1016/j.techfore.2022.12168> 2

Chen, J., Taylor, J. E., & Wei, H.-H. (2012). Modeling building occupant network energy consumption decision-making: The interplay between network structure and conservation. Energy and Buildings, 47, 515–524. <https://doi.org/10.1016/j.enbuild.2011.12.026>

Chen, R., Fan, R., Yao, Q., & Qian, R. (2023). Evolutionary dynamics of homeowners’ energy-efficiency retrofit decision-making in complex network. Journal of Environmental Management, 326, 116849. <https://doi.org/10.1016/j.jenvman.2022.116849>

Chen, S., Zhang, H., Guan, J., & Rao, Z. (2020). Agent-based modeling and simulation of stochastic heat pump usage behavior in residential communities. Building Simulation, 13(4), 803–821. <https://doi.org/10.1007/s12273-020-0625-2>

Danielis, R., Scorrano, M., Massi Pavan, A., & Blasuttigh, N. (2023). Simulating the Diffusion of Residential Rooftop Photovoltaic, Battery Storage Systems and Electric Cars in Italy. An Exploratory Study Combining a Discrete Choice and Agent-Based Modelling Approach. Energies, 16(1), 557. <https://doi.org/10.3390/en16010557>

de Wildt, T. E., Boijmans, A. R., Chappin, E. J. L., & Herder, P. M. (2021). An ex ante assessment of value conflicts and social acceptance of sustainable heating systems: An agent-based modelling approach. Energy Policy, 153, 112265. <https://doi.org/10.1016/j.enpol.2021.112265>

de Wildt, T. E., Chappin, E. J. L., van de Kaa, G., Herder, P. M., & van de Poel, I. R. (2020). Conflicted by decarbonisation: Five types of conflict at the nexus of capabilities and decentralised energy systems identified with an agent-based model. Energy Research & Social Science, 64, 101451. <https://doi.org/10.1016/j.erss.2020.101451>

Derkenbaeva, E., Hofstede, G. J., van Leeuwen, E., & Halleck Vega, S. (2023). Simulating households’ energy transition in Amsterdam: An agent-based modeling approach. Energy Conversion and Management, 294, 117566. <https://doi.org/10.1016/j.enconman.2023.117566>

Ebrie, A. S., & Kim, Y. J. (2022). Investigating Market Diffusion of Electric Vehicles with Experimental Design of Agent-Based Modeling Simulation. Systems, 10(2), 28. <https://doi.org/10.3390/systems10020028>

Egner, L., & Klöckner, C. (2022). Effect of Policy Implementation on Energy Retrofit Behavior and Energy Consumption in a Simulated Neighborhood. Journal of Artificial Societies and Social Simulation, The, 25, 2022. <https://doi.org/10.18564/jasss.4936>

Faber, A., Valente, M., & Janssen, P. (2010). Exploring domestic micro-cogeneration in the Netherlands: An agent-based demand model for technology diffusion. Energy Policy, 38(6), 2763–2775. <https://doi.org/10.1016/j.enpol.2010.01.008>

Fouladvand, J., Ghorbani, A., Sarı, Y., Hoppe, T., Kunneke, R., & Herder, P. (2022). Energy security in community energy systems: An agent-based modelling approach. Journal of Cleaner Production, 366, 132765. <https://doi.org/10.1016/j.jclepro.2022.132765>

Friege, J. (2016). Increasing homeowners’ insulation activity in Germany: An empirically grounded agent-based model analysis. Energy and Buildings, 128, 756–771. <https://doi.org/10.1016/j.enbuild.2016.07.042>

Golmaryami, S., Nunes, M. L., & Ferreira, P. (2024). The role of social learning on consumers’ willingness to engage in demand-side management: An agent-based modelling approach. Smart Energy, 14, 100138. <https://doi.org/10.1016/j.segy.2024.100138>

Hicks, A. L., & Theis, T. L. (2014). An agent based approach to the potential for rebound resulting from evolution of residential lighting technologies. The International Journal of Life Cycle Assessment, 19(2), 370–376. <https://doi.org/10.1007/s11367-013-0643-8>

Hidayatno, A., Jafino, B. A., Setiawan, A. D., & Purwanto, W. W. (2020). When and why does transition fail? A model-based identification of adoption barriers and policy vulnerabilities for transition to natural gas vehicles. Energy Policy, 138, 111239. <https://doi.org/10.1016/j.enpol.2020.111239>

Hoffmann, S., Adelt, F., & Weyer, J. (2020). Modelling End-User Behavior and Behavioral Change in Smart Grids. An Application of the Model of Frame Selection. Energies, 13(24), 6674. <https://doi.org/10.3390/en13246674>

Huang, P., Lovati, M., Shen, J., Chai, J., & Zhang, X. (2022). Investigation of the Peer-to-Peer energy trading performances in a local community under the future climate change scenario in Sweden. Energy Reports, 8, 989–1001. <https://doi.org/10.1016/j.egyr.2021.12.032>

Jagadish, A., Dwivedi, P., McEntire, K. D., & Chandar, M. (2019). Agent-based modeling of “cleaner” cookstove adoption and woodfuel use: An integrative empirical approach. Forest Policy and Economics, 106, 101972. <https://doi.org/10.1016/j.forpol.2019.101972>

Jensen, T., Holtz, G., Baedeker, C., & Chappin, É. J. L. (2016). Energy-efficiency impacts of an air-quality feedback device in residential buildings: An agent-based modeling assessment. Energy and Buildings, 116, 151–163. <https://doi.org/10.1016/j.enbuild.2015.11.067>

Jensen, T., Holtz, G., & Chappin, É. J. L. (2015). Agent-based assessment framework for behavior-changing feedback devices: Spreading of devices and heating behavior. Technological Forecasting and Social Change, 98, 105–119. <https://doi.org/10.1016/j.techfore.2015.06.006>

Khansari, N., & Hewitt, E. (2020). Incorporating an agent-based decision tool to better understand occupant pathways to GHG reductions in NYC buildings. Cities, 97, 102503. <https://doi.org/10.1016/j.cities.2019.102503>

Kotthoff, F., & Hamacher, T. (2022). Calibrating Agent-Based Models of Innovation Diffusion with Gradients. Journal of Artificial Societies and Social Simulation, 25. <https://doi.org/10.18564/jasss.4861>

Kowalska-Pyzalska, A., Maciejowska, K., Suszczyński, K., Sznajd-Weron, K., & Weron, R. (2014). Turning green: Agent-based modeling of the adoption of dynamic electricity tariffs. Energy Policy, 72, 164–174. <https://doi.org/10.1016/j.enpol.2014.04.021>

Lee, M., & Hong, T. (2019). Hybrid agent-based modeling of rooftop solar photovoltaic adoption by integrating the geographic information system and data mining technique. Energy Conversion and Management, 183, 266–279. <https://doi.org/10.1016/j.enconman.2018.12.096>

Lee, Y. S., & Malkawi, A. M. (2014). Simulating multiple occupant behaviors in buildings: An agent-based modeling approach. Energy and Buildings, 69, 407–416. <https://doi.org/10.1016/j.enbuild.2013.11.020>

Liu, Y., Xu, X., Liu, Y., Liu, J., Hu, W., Yang, N., Jawad, S., & Wei, Z. (2024). Scenario-based operation of an integrated rural multi-energy system considering agent-based farmer-behavior modeling. Energy Conversion and Management, 304, 118204. <https://doi.org/10.1016/j.enconman.2024.118204>

Lovati, M., Huang, P., Olsmats, C., Yan, D., & Zhang, X. (2021). Agent Based Modelling of a Local Energy Market: A Study of the Economic Interactions between Autonomous PV Owners within a Micro-Grid. Buildings, 160. <https://doi.org/10.3390/buildings11040160>

Madler, J., Harding, S., & Weibelzahl, M. (2023). A multi-agent model of urban microgrids: Assessing the effects of energy-market shocks using real-world data. Applied Energy, 343, 121180. <https://doi.org/10.1016/j.apenergy.2023.121180>

Maqbool, A. S., Baetens, J., Lotfi, S., Vandevelde, L., & Van Eetvelde, G. (2019). Assessing financial and flexibility incentives for integrating wind energy in the grid via agent-based modeling. Energies, 12(22), 4314. <https://www.mdpi.com/1996-1073/12/22/4314>

McCoy, D., & Lyons, S. (2014). Consumer preferences and the influence of networks in electric vehicle diffusion: An agent-based microsimulation in Ireland. Energy Research & Social Science, 3, 89–101. <https://doi.org/10.1016/j.erss.2014.07.008>

Meles, T. H., & Ryan, L. (2022). Adoption of renewable home heating systems: An agent-based model of heat pumps in Ireland. Renewable and Sustainable Energy Reviews, 169, 112853. <https://doi.org/10.1016/j.rser.2022.112853>

Moglia, M., Nygaard, C., Glackin, S., Cook, S., & Tapsuwan, S. (2022). Hybrid Approach for Modelling the Uptake of Residential Solar PV Systems, with Case Study Application in Melbourne, Australia. Journal of Artificial Societies and Social Simulation, The, 25, 2. <https://doi.org/10.18564/jasss.4921>

Muelder, H., & Filatova, T. (2018). One Theory - Many Formalizations: Testing Different Code Implementations of the Theory of Planned Behaviour in Energy Agent-Based Models. Journal of Artificial Societies and Social Simulation, 21(4), 5. <https://jasss.soc.surrey.ac.uk/21/4/5.html>

Mueller, M. G., & de Haan, P. (2009). How much do incentives affect car purchase? Agent-based microsimulation of consumer choice of new cars—Part I: Model structure, simulation of bounded rationality, and model validation. Energy Policy, 37(3), 1072–1082. <https://doi.org/10.1016/j.enpol.2008.11.002>

Mussawar, O., Urs, R. R., Mayyas, A., & Azar, E. (2023). Performance and prospects of urban energy communities conditioned by the built form and function: A systematic investigation using agent-based modeling. Sustainable Cities and Society, 99, 104957. <https://doi.org/10.1016/j.scs.2023.104957>

Nägeli, C., Jakob, M., Catenazzi, G., & Ostermeyer, Y. (2020). Towards agent-based building stock modeling: Bottom-up modeling of long-term stock dynamics affecting the energy and climate impact of building stocks. Energy and Buildings, 211, 109763. <https://doi.org/10.1016/j.enbuild.2020.109763>

Nava-Guerrero, G.-C., Hansen, H. H., Korevaar, G., & Lukszo, Z. (2021). The effect of group decisions in heat transitions: An agent-based approach. Energy Policy, 156, 112306. <https://doi.org/10.1016/j.enpol.2021.112306>

Nava-Guerrero, G.-C., Hansen, H. H., Korevaar, G., & Lukszo, Z. (2022). An agent-based exploration of the effect of multi-criteria decisions on complex socio-technical heat transitions. Applied Energy, 306, 118118. <https://doi.org/10.1016/j.apenergy.2021.118118>

Neshat, N., Kaya, M., & Ghaboulian Zare, S. (2023). Exploratory policy analysis for electric vehicle adoption in European countries: A multi-agent-based modelling approach. Journal of Cleaner Production, 414, 137401. <https://doi.org/10.1016/j.jclepro.2023.137401>

Noori, M., & Tatari, O. (2016). Development of an agent-based model for regional market penetration projections of electric vehicles in the United States. Energy, 96, 215–230. <https://doi.org/10.1016/j.energy.2015.12.018>

Nurwidiana, N., Sopha, B., & Widyaparaga, A. (2022). Simulating Socio-Technical Transitions of Photovoltaics Using Empirically Based Hybrid Simulation-Optimization Approach. Sustainability, 14, 5411. <https://doi.org/10.3390/su14095411>

Opiyo, N. N. (2019). Impacts of neighbourhood influence on social acceptance of small solar home systems in rural western Kenya. Energy Research & Social Science, 52, 91–98. <https://doi.org/10.1016/j.erss.2019.01.013>

Palmer, J., Sorda, G., & Madlener, R. (2015). Modeling the diffusion of residential photovoltaic systems in Italy: An agent-based simulation. Technological Forecasting and Social Change, 99, 106–131. <https://doi.org/10.1016/j.techfore.2015.06.011>

Rai, V., & Robinson, S. A. (2015). Agent-based modeling of energy technology adoption: Empirical integration of social, behavioral, economic, and environmental factors. Environmental Modelling & Software, 70, 163–177. <https://doi.org/10.1016/j.envsoft.2015.04.014>

Schiera, D., Minuto, F. D., Bottaccioli, L., Borchiellini, R., & Lanzini, A. (2019). Analysis of Rooftop Photovoltaics Diffusion in Energy Community Buildings by a Novel GIS- and Agent-Based Modeling Co-Simulation Platform. IEEE Access, 7, 93404–93432. <https://doi.org/10.1109/ACCESS.2019.2927446>

Shen, M., Li, X., Lu, Y., Cui, Q., & Wei, Y.-M. (2021). Personality-based normative feedback intervention for energy conservation. Energy Economics, 104, 105654. <https://doi.org/10.1016/j.eneco.2021.105654>

Silvia, C., & Krause, R. M. (2016). Assessing the impact of policy interventions on the adoption of plug-in electric vehicles: An agent-based model. Energy Policy, 96, 105–118. <https://doi.org/10.1016/j.enpol.2016.05.039>

Snape, J. R., Boait, P. J., & Rylatt, R. M. (2015). Will domestic consumers take up the renewable heat incentive? An analysis of the barriers to heat pump adoption using agent-based modelling. Energy Policy, 85, 32–38. <https://doi.org/10.1016/j.enpol.2015.05.008>

Sopha, B. mAYA, Klöckner, C. A., & Hertwich, E. G. (2011). Exploring policy options for a transition to sustainable heating system diffusion using an agent-based simulation. Energy Policy, 39(5), 2722–2729. <https://doi.org/10.1016/j.enpol.2011.02.041>

Stavrakas, V., Papadelis, S., & Flamos, A. (2019). An agent-based model to simulate technology adoption quantifying behavioural uncertainty of consumers. Applied Energy, 255, 113795. <https://doi.org/10.1016/j.apenergy.2019.113795>

Sun, Y., Silva, E., Tian, W., Choudhary, R., & Leng, H. (2018). An Integrated Spatial Analysis Computer Environment for Urban-Building Energy in Cities. Sustainability, 10, 4235. <https://doi.org/10.3390/su10114235>

Tian, S., Lu, Y., Ge, X., & Zheng, Y. (2021). An agent-based modeling approach combined with deep learning method in simulating household energy consumption. Journal of Building Engineering, 43, 103210. <https://doi.org/10.1016/j.jobe.2021.103210>

van der Kam, M., Peters, A., van Sark, W., & Alkemade, F. (2019). Agent-Based Modelling of Charging Behaviour of Electric Vehicle Drivers. Journal of Artificial Societies and Social Simulation, 22(4), 7. <https://jasss.soc.surrey.ac.uk/22/4/7.html>

Walzberg, J., Dandres, T., Merveille, N., Cheriet, M., & Samson, R. (2019). Assessing behavioural change with agent-based life cycle assessment: Application to smart homes. Renewable and Sustainable Energy Reviews, 111, 365–376. <https://doi.org/10.1016/j.rser.2019.05.038>

Wang, H., Fang, H., Yu, X., & Liang, S. (2018). How real time pricing modifies Chinese households’ electricity consumption. Journal of Cleaner Production, 178, 776–790. <https://doi.org/10.1016/j.jclepro.2017.12.251>

Weron, T., Kowalska-Pyzalska, A., & Weron, R. (2018). The role of educational trainings in the diffusion of smart metering platforms: An agent-based modeling approach. Physica A: Statistical Mechanics and Its Applications, 505, 591–600. <https://doi.org/10.1016/j.physa.2018.03.086>

Williams, B., Bishop, D., Hooper, G., & Chase, J. G. (2024). Driving change: Electric vehicle charging behavior and peak loading. Renewable and Sustainable Energy Reviews, 189, 113953. <https://doi.org/10.1016/j.rser.2023.113953>

Wolf, I., Schröder, T., Neumann, J., & de Haan, G. (2015). Changing minds about electric cars: An empirically grounded agent-based modeling approach. Technological Forecasting and Social Change, 94, 269–285. <https://doi.org/10.1016/j.techfore.2014.10.010>

Yue, T., Long, R., Chen, H., Liu, J., Liu, H., & Gu, Y. (2020). Energy-saving behavior of urban residents in China: A multi-agent simulation. Journal of Cleaner Production, 252, 119623. <https://doi.org/10.1016/j.jclepro.2019.119623>

Zhang, H., Vorobeychik, Y., Letchford, J., & Lakkaraju, K. (2016). Data-driven agent-based modeling, with application to rooftop solar adoption. Autonomous Agents and Multi-Agent Systems, 30(6), 1023–1049. <https://doi.org/10.1007/s10458-016-9326-8>

Zhang, N., Lu, Y., Chen, J., & Hwang, B.-G. (2022). An agent-based diffusion model for Residential Photovoltaic deployment in Singapore: Perspective of consumers’ behaviour. Journal of Cleaner Production, 367, 132793. <https://doi.org/10.1016/j.jclepro.2022.132793>

Zhang, Z., & Han, Z. (2024). Exploring coevolution in the diffusion of green products between consumers and enterprises—An agent-based model of two-layer heterogeneous networks. Journal of Cleaner Production, 450, 141689. <https://doi.org/10.1016/j.jclepro.2024.141689>
