# Supplementary material for: Bridging behavioral theory and household energy decisions: enhancing agent-based models with behavioral analysis
Source: Front Psychol. 2025 Jul 9;16:1568730. doi: 10.3389/fpsyg.2025.1568730 (PMC12284503; doi:10.3389/fpsyg.2025.1568730)

**Appendix B – Theories of behavior formalized in our data set.**

**Theory of Planned behavior (TPB)**

The TPB (Ajzen, 1991) is an extension of the theory of reasoned action (TRA), which was developed in 1967 and re-examined in 2000 (Ajzen & Fishbein, 2000). The TRA explains how people form a behavioral intention, which leads to the behavior itself. The key components of the TRA are attitudes and subjective norms. According to the theory, attitudes are shaped by behavioral belief (based on assigned consequences of the behavior) and the evaluation of these consequences. Subjective norms are shaped by normative beliefs (based on which behaviors would be approved by relevant others) and the motivation to comply with social pressure. The TPB adds the concept of perceived behavioral control (PCB) to the TRA: if the behavior is perceived to be easy or difficult to perform. The PCB is shaped by a person’s control beliefs, which involve the set of perceptions of factors that may facilitate or hinder the behavior. Empirical work on the theory shows that background factors (e.g., personality traits or available information) influence the three main concepts of the theory. Figure **A** shows an overview.

**Stage model of self-regulated behavioral change (SMBC)**

The SMBC (Bamberg, 2013) stems from the model of action phases (Gollwitzer, 1990), in which behavior is understood to be a *‘temporal, horizontal path starting with a person's desires and ending with the evaluation of the achieved action outcome*’ (ibid.). The SMBC assumes that this temporal path consists of four subsequent and independent stages (pre-decisional, pre-actional, actional, and post-actional) in which individuals perform different tasks. In the first stage, individuals reflect deliberately on competing motivations and commit to the behavior change by forming a goal intention. In the second stage, several different behavioral strategies are weighed and chosen, resulting in a behavioral intention. In the third stage, the behavioral strategy is initiated and facilitated by forming an implementation intention, which is more specific than the behavioral intention, on when and where the behavior is executed. In the fourth state, desired outcomes are compared with achieved outcomes, and the individual might struggle with temptations that hinder achieving the desired outcomes. Figure **B** shows an overview.

**Unified theory of acceptance and use of technology (UTAUT)**

The UTAUT (Venkatesh et al., 2003) was first used to explain the use of information technology and merge concepts from eight different behavioral theories. Just like the TPB and the SSCB, the UTAUT states that behavioral intentions are a precursor to behavior. The UTAUT holds four key constructs that influence intention (performance expectancy, effort expectancy, social influence, facilitation conditions) and defines background factors (age, gender, experience, and voluntariness of use -if the use of the innovation is perceived as being out of free will). Performance expectancy is the degree to which someone believes that the use of an innovation will have benefits or downsides. Effort expectancy is associated with the degree to which someone believes it is hard or easy to use the innovation. Social influence in this theory is the degree to which someone believes relevant others think he or she should use the innovation. Facilitating conditions are defined as the belief that organizational and technical infrastructure are in place to support the use of innovation. Gender, age, experience, and voluntariness of use are posited to moderate the impact of the four key constructs on behavioral intention. Figure **C** shows an overview.

**Model of frame selection (MFS)**

The MFS (Esser & Kroneberg, 2015) holds the notions of ‘definition of the situation’ and ‘variable rationality’ as key concepts. The theory is called the model of frame selection because the starting point is how an individual defines, or frames, a situation (“what kind of situation is this?”). After the situation is framed, a script is chosen. This means that the actions that a person is willing to take are activated (“which way of acting is appropriate?”). This, in turn, results in action (“what am I going to do?”). The selection of frame and script are governed by a mode of rationality, ranging from reflective to automatic. The theory stipulates that the presence of situational objects (opportunity, motivation, effort, and accessibility) influences which mode of rationality is chosen. Figure **D** shows an overview.

**Consumat**

The Consumat framework is introduced by Jager et al. (2000) and describes consumer behavior. It combines driving forces at both the macro level (human environments like, for example, culture, technology, and demographics) and the micro level (individual strategies for behavior change). The micro level contains consumers’ needs, the opportunities that they have, their ability to react to opportunities, and an assessment of how certain they are about behavioral outcomes. The framework also contains the concept of memory (it is easier to perform the behavior when it has already been performed before) and a behavioral control function (as explained in the TPB section). Depending on high or low values of each of these factors, four different modes of cognitive processes are selected to determine an individual’s choice. In the deliberate mode, people assess the consequences of all possible decisions. The social comparison and imitation mode allow for comparisons of one's own behavior with that of others, and in the repetition mode, individuals simply repeat what they did before. Figure **E** shows an overview.

**Figure A.** Schematic overview of the TPB, from <https://www.sciencedirect.com/topics/medicine-and-dentistry/theory-of-planned-behavior>


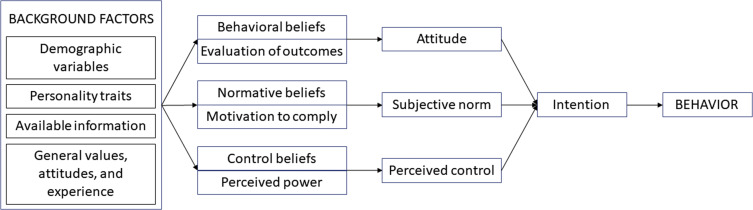


**Figure B**. Schematic overview of SSBC, from <https://www.sciencedirect.com/science/article/pii/S0272494412000655#bib2>


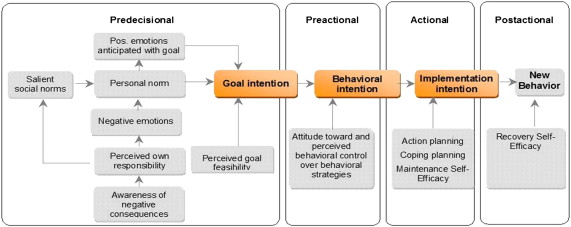


**Figure C. Figure C**. Schematic overview of UTAUT, from Venkatesh et al., 2003


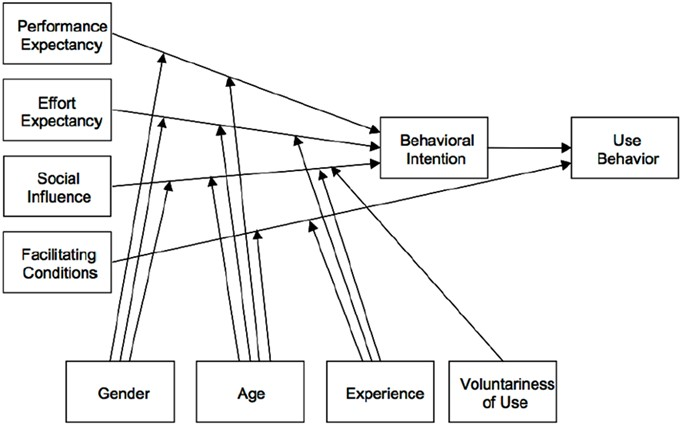


**Figure D**. Schematic overview of the MFS. <https://www.kroneberg.eu/model-of-frame-selection/>
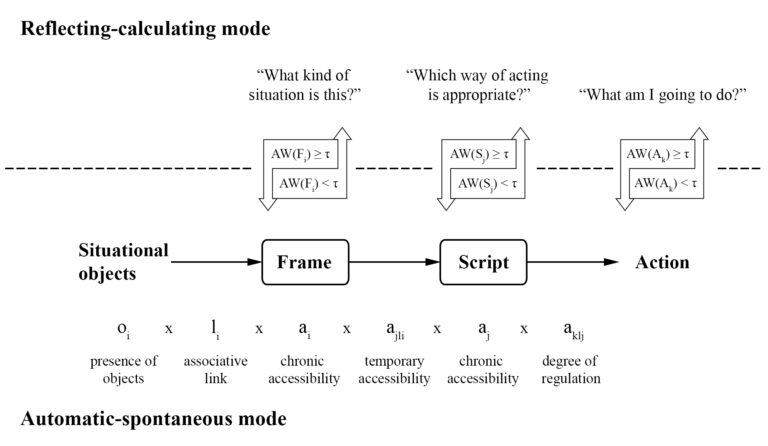


**Figure E.** Overview of the Consumat framework <https://www.sciencedirect.com/science/article/pii/S0921800900002202>


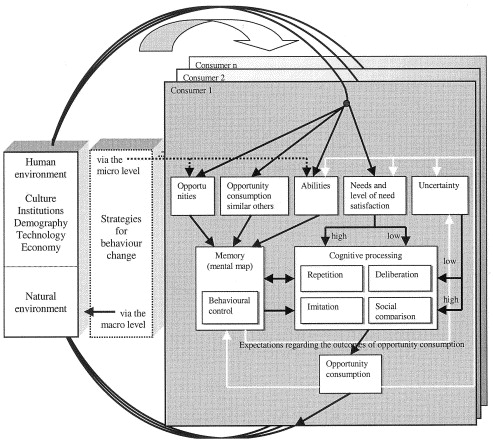

Supplement: Supplementary file 2 [file Supplementary_file_2.docx]
